# The Effect of Electronic Structure on the Phases Present in High Entropy Alloys

**DOI:** 10.1038/srep39803

**Published:** 2017-01-06

**Authors:** Zhaoyuan Leong, Jan S. Wróbel, Sergei L. Dudarev, Russell Goodall, Iain Todd, Duc Nguyen-Manh

**Affiliations:** 1 Department of Materials Science & Engineering, University of Sheffield, Sheffield, UK; 2 CCFE, Culham Science Centre for Fusion Energy, Culham, Abingdon, OX14 3DB, Oxfordshire, UK; 3 Faculty of Materials Science and Engineering, Warsaw University of Technology, ul. Wołoska 141, 02-507 Warszawa, Poland

## Abstract

Multicomponent systems, termed High Entropy Alloys (HEAs), with predominantly single solid solution phases are a current area of focus in alloy development. Although different empirical rules have been introduced to understand phase formation and determine what the dominant phases may be in these systems, experimental investigation has revealed that in many cases their structure is not a single solid solution phase, and that the rules may not accurately distinguish the stability of the phase boundaries. Here, a combined modelling and experimental approach that looks into the electronic structure is proposed to improve accuracy of the predictions of the majority phase. To do this, the Rigid Band model is generalised for magnetic systems in prediction of the majority phase most likely to be found. Good agreement is found when the predictions are confronted with data from experiments, including a new magnetic HEA system (CoFeNiV). This also includes predicting the structural transition with varying levels of constituent elements, as a function of the valence electron concentration, *n,* obtained from the integrated spin-polarised density of states. This method is suitable as a new predictive technique to identify compositions for further screening, in particular for magnetic HEAs.

In the continuing search for new alloys, a recent trend has been to move away from systems dominated by a single element with minor additions towards alloys consisting of multiple elements in similar quantities. These alloys were expected to show multiple complex phases (defined in this work as non-simple structures; FCC, BCC and HCP structures are considered to be simple phases), and hence be of limited use, but experimental evidence has shown that some such alloys, called High Entropy Alloys (HEAs)^1^,^2^,^3^,^4^, frequently form simple phases. They were first observed in the five-component equiatomic alloy CoCrFeNiMn (here denoted CCFN-Mn_1.0_, as the base composition CoCrFeNi, CCFN occurs widely in these alloys)^1^ forming a face-centred cubic (FCC) phase. Further experiments suggested that such phases resisted ordering^2^, which was attributed to stabilisation by chemical and magnetic contributions to the entropy resulting from the multiple components^5^,^6^ (although this is disputed as the sole, or even primary, cause^7^). Investigation of the properties of such alloys highlighted a number of attractive features (such as high strength combined with ductility) which makes the design of alloys using the effect, whatever the cause, desirable^4^.

Most of the experimental studies of these alloys make use of processing methods, such as arc melting, likely to give non-equilibrium conditions for phase formation. More recent studies of some alloys have found that many are not simply single phase, or stable against transformation. For example, it has been found that on heat treatment, additional complex phases form in CCFN-Mn_1.0_^8^, and that other alloys, are not homogenous on the atomic scale and perfect solid solutions are not obtained^9^,^10^. However, the simple phase HEAs appear to retain their structures from the as-cast condition as a majority phase when annealed below a defined temperature, unless a) heat treated for extensive durations (500 h) leading to the appearance of grain-boundary precipitates^8^,^11^, or b) the composition is subjected to severe plastic deformation^12^. This metastability may reflect the near-ideal solid solution nature of simple phase HEAs^7^, and furthermore may lead to local segregation in the short range order^13^. X-ray diffraction peaks of the simple FCC phase in CCFN and CCFN-Pd_x_ compositions have been observed by Dahlborg *et al*. to decompose into multiple components^14^ following heat treatment, as hypothesised in previous studies^13^,^15^. As such, in this work, we will follow Dahlborg *et al*.’s^14^ suggested definition of HEAs as multicomponent alloys presenting simple diffraction patterns corresponding to FCC, BCC, or HCP structures, either as a single phase or multiple phases with very close lattice parameters. These may adopt a complex phase as the compositions deviate further from the ideal solid solution^8^,^11^.

Many attempts have been made to discriminate between compositions for which simple phases apparently occur and those where they do not. Empirical rules have met with some success in determining the phases of these metastable structures, including utilising the Hume-Rothery rules^16^ to relate the Valence Electron Concentration (VEC) to the phase formed^17^, and the Miedema model to investigate solid-solution stability^18^. Using Principal Component Analysis (PCA), Dominguez *et al*.^19^ analysed the contributions of five thermodynamic and electronic variables (enthalpy of mixing, entropy of mixing, atomic size mismatch, electronegativity difference, and VEC) and found that different simple structures and intermetallic compounds are reasonably well differentiated by the values of VEC and the enthalpy of mixing of the alloy; other factors identified in previous work^17^,^19^,^20^ such as the electronegativity difference, atomic size difference, and the configurational entropy of solid solutions were found to have a lesser influence on phase stability.

Although this framework offers differentiation between the simple phases FCC and BCC, the occurrence of simple and complex phases is not discriminated, and the accuracy of these empirical rules appears to be strongly dependent on the amount of alloying additions^17^, limiting the extent to which alloy compositions can be designed. The solution may be to consider the electronic structure, the effects of which are not fully captured by the empirical approaches. To access such information, the Density Functional Theory (DFT) formalism^21^ can be used, such as in investigating enthalpies^22^ and entropies^23^ of formation for HEAs within spin-polarised electronic structure calculations. One simplification of the DFT approach is the Rigid Band Approximation (RBA), originally proposed for non-magnetic metallic alloys, which assumes that the energy difference between two phases is given entirely by the difference in band-structure energy^24^,^25^,^26^,^27^. This allows the study of the structural energy difference, and hence prediction of the most stable phase, in a computationally less intense manner than full DFT calculations. It has been successfully implemented for different alloy systems^24^,^25^,^26^,^27^, though not previously for HEAs. The RBA models are simple enough to readily interpret the available experimental data yet powerful enough to correctly predict the new stable phase in a multicomponent system.

In order to better understand, and improve on, the phase discrimination found in 2-dimensional plots of the empirical models^19^, we experimentally investigate the transition from simple to complex phases of a number of 5-component HEA alloys based on the equiatomic CoCrFeNi (CCFN) composition through X-Ray Diffraction (XRD) characterisation. The compositions are compared to predictions from the RBA approach, in particular of the transition between phases that occurs with changing stoichiometry. We generalise the original RBA approach^19^,^20^ to include the effects of magnetism using the Stoner model^28^,^29^,^30^,^31^,^32^, as this affects the difference in energy between two phases (particularly since the constituent metals Co, Cr, Fe, and Ni already exhibit magnetic behaviour; other CoCrFeNi-type alloys have also been reported for their magnetic properties^23^,^33^,^34^,^35^). It is found that the VEC parameter is able to serve as a good predictor of simple-complex transitions when the s, p, and d valence electrons are accounted for. Therefore the RBA model can be used as a simple but relatively accurate method based on electronic structure calculations for phase stability prediction in HEAs as will be demonstrated within this paper, in particular in the understanding and design of alloys in the new CoFeNiV system.

## Results

### Experimental Identification of Phases Adopted by CoCrFeNi-type Compositions

20 HEA compositions based on CoCrFeNiA_X_, where A = Pd, Al, V, and Ti (henceforth denoted as CCFN-Pd_x_, CCFN-Al_x_, CCFN-V_x_, and CCFN-Ti_x_) with nominal compositions given in Table 1, were made. The amount of each addition was selected to explore the transition between simple (FCC and BCC) and complex (B2, Sigma, and C14) phases. We have extended the analysis of CCFN-A_x_ compositions beyond those previously reported to identify the accuracy of the RBA-predicted phase stability in terms of the relative behaviour of the electronic densities of states.

Further compositions from the novel CoFeNiV_x_ system (henceforth denoted CoFN-V_x_) were also prepared in order to validate the use of the RBA model for unknown compositions against experimental data. The compositional variations are tabulated in Table 1.

To analyse the phases adopted by these compositions, XRD characterisation experiments were performed. The XRD patterns shown here are for ease of reference; for detailed patterns showing the Rietveld refinement curves and corresponding residuals, the reader may refer to the supplementary information. Visual comparison of the calculated phase indicates that the selected phases are in agreement with the observed patterns. The XRD patterns show that, within detection limits, the FCC phase is present for compositions CCFN-Pd_0.5_, CCFN-Pd_1.0_, and CCFN-Pd_1.5_. For the CCFN-Al_x_ family the FCC phase is maintained for CCFN-Al_0.5_ with some small amounts of BCC/B2 formation. The BCC phase is fully adopted at the CCFN-Al_1.0_ composition, with the BCC/B2 phases at CCFN-Al_3.0_. Similarly, the CCFN-V_x_ family shows FCC alone at the smaller V additions of CCFN-V_0.5_ and CCFN-V_0.8_ with some secondary phase formation for larger V additions. A mixed phase is observed for CCFN-V_1.0_ and the composition fully adopts the Sigma phase by CCFN-V_2.0_. The phase formation and Rietveld refined lattice parameters are summarised in Table 1; where increasing amounts of Pd, Al, and V are added to CCFN there is an associated increase in the lattice parameter. Although XRD peak asymmetry may be indicative of decomposition of the solid solution, the origin of this decomposition is the subject of ongoing research^13^,^14^. In this work, we make the assumption that the compositions exhibiting CCFN simple phase(s) are in a random solid solution, albeit that these may experience segregation in the short-range order^13^.

Phase discrimination in a 2-dimensional plot of the enthalpy of mixing against VEC was first presented by Dominguez *et al*.^14^, where a single BCC phase was identified for 3 < VEC < 6, a single FCC phase for 8 < VEC < 11, and complex phases at intermediate values (for 6 < VEC < 8). The empirical VEC for an alloy may be obtained from the weighted average of the electrons accommodated in the s, p, d orbitals of the alloy’s constituent elements^17^. Our experimental results show that the compositions of complex phases (*c.f.* Table 1) indeed possess values in the intermediate range of 6 < VEC < 7.8. The cubic B2 presence in CCFN-Al_x_ compositions determined experimentally is observed fully only from VEC ≤ 6.81; the tetragonal Sigma phase presence in the CCFN-V_x_ compositions at VEC ≤ 7.77; and the hexagonal C14 phase presence in CCFN-Ti_x_ compositions at VEC ≤ 7.4.

These observations highlight the change in phases that can result from the addition of alloying elements to the CCFN composition which are quantified by values of the semi-empirical VEC. The RBA method employed in this study allows investigation of the relative structural stability of CCFN (CCFN-A_x_) as a function of the exact valence electron concentration, *n,* which is, in turn, obtained from the integration of the density of electronic states (*cf.* equation (2) below). *n* should thus be able to better describe the various phase changes as the electronic structure can now be accounted for.

### Background of the RBA analysis

Within DFT, the total energy difference between two structures (1 and 2) for a particular alloy at a fixed volume can be decomposed into two contributions^24^,^36^,^37^:


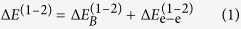


where the first contribution represents the change in band energy between the two structures, and the second contribution arises from the structural energy difference in electrostatic and electron-electron interactions. By using the RBA/frozen potential approach^36^,^37^, the energy difference between two non-magnetic phases can be simply evaluated by comparing the band-structure energy difference computed using the same frozen potential for the two structures at a fixed volume. This approximation is valid to the first order, not only for an elemental metal, but also for metallic alloys where the second contribution is considered to be small^24^,^25^,^26^,^27^. In order to analyse the phase stability over a wide range of concentrations the energy difference can be presented as a function of *n*, which can in turn be determined from the integration of the total electronic density of states (DOS) per atom up to the Fermi energy.

For magnetic alloys, such as CCFN-based HEAs investigated in this work, applying the Stoner model to the RBA allows the band energy difference to be decomposed to contributions from the spin-polarised band energy and the double-counting contributions arising from magnetic interactions^28^,^29^,^31^,^32^. In this case, the value of valence electron concentration, *n,* can be obtained from the integration of the spin-polarised total DOS per atom as


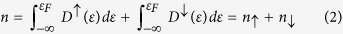


where *D*^↑^(*ε*) and *D*^↓^(*ε*) are the spin-polarised total DOS per atom for electrons with spin-up and spin-down, respectively, and *ε*_*F*_ is the Fermi energy.

**Figure 1:**
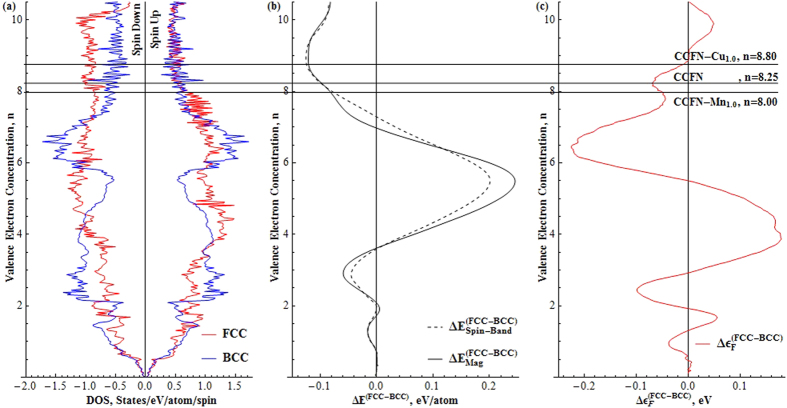
(**a**) Spin-polarised density of states of FCC and BCC 32-atom Special Quasi-Random Structure (SQS) CCFN and; (**b**) Spin-polarised band energy difference between FCC and BCC CCFN (i) Without double-counting Stoner corrections, (ii) With Stoner Correction and; (**c**) The Fermi energy difference between the FCC and BCC structure (*c.f.* equation (4)).

Exact application of the Stoner model requires knowledge of magnetic moments of all atoms in the system but it can be simplified through using an effective Stoner parameter, *I*_eff_. This is defined as the exchange splitting of the on-site energies of electrons with spin-up and spin-down due to average atomic magnetic moment, *m*_*av*_, which can be obtained from the non-magnetic total DOS and the value of the average atomic magnetic moment of the entire simulation cell. The energy difference between any two magnetic structures can be thus written as:


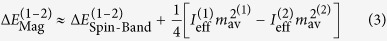


where the first term is related to the spin-polarised band energy difference obtained from the total DOS per atom and per electron spin and the second term is the double-counting contribution coming from magnetic interactions. As shown in Supplementary Table S1, the energy differences calculated with a knowledge of magnetic moments of all atoms in the system and those obtained using average magnetic moments and effective Stoner parameters are in quantitative agreement and are in line with the results obtained using the LMTO code^38^. Thus, for the RBA analysis of stability of other CCFN-based alloys the simplified method based on the effective Stoner parameter will be applied. The derivation of the RBA for magnetic systems using the Stoner model can be found in the supplementary information [see equations (I–V)]. Figure 1(a) shows the spin-polarised DOS of disordered simple FCC and BCC phases calculated utilising a Special Quasi-random Structure (SQS)^39^,^40^ generated Co_8_Cr_8_Fe_8_Ni_8_ structure, representing the CCFN composition, which is used for the RBA analysis. Figure 1(b) shows the resulting FCC-BCC band energy difference of CCFN alloys calculated with and without Stoner corrections as a function of *n*. The lines drawn in Fig. 1b were obtained by the Eq. (3) in which the first contribution is determined by Equation (V) and the second one, by Eq. (VI)–(VII) in the Supplementary Information. The spin-polarised density of states for FCC (1) and BCC structures (2) shown in Fig. 1a have been used as input for calculating the integrals in Equations (V) and (VII), for the spin-polarised band energy difference and average magnetic moment, respectively. It is important to note that the above procedure can be generalised to analyse the relative stability for more than two structures^24^,^25^,^41^, as will be shown in the following Section.

### RBA Phase Stability as a Function of the Valence Electron Concentration

**Figure 2:**
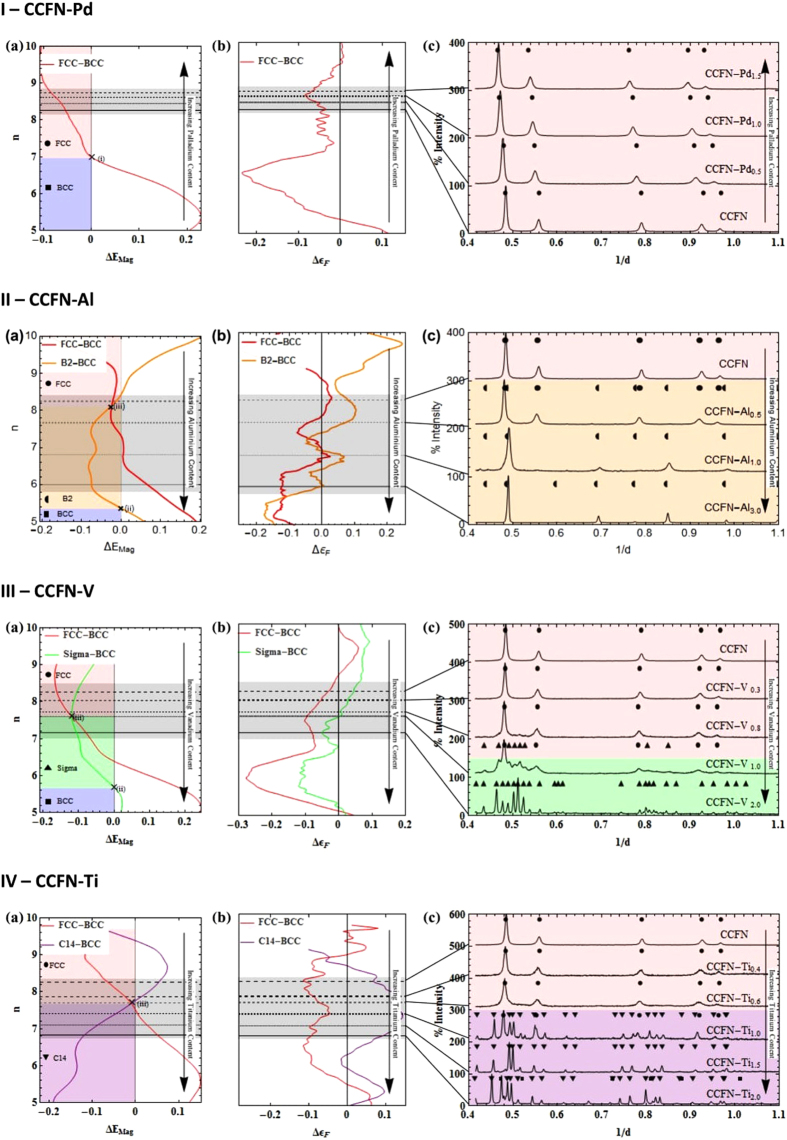
Predictions and experimental results for several alloy systems, I – CCFN-Pd, II – CCFN-Al, III –CCFN-V, and IV – CCFN-Ti, showing for each, the change in phase stability with increasing 5^th^ element content through (**a**) VEC, *n*, against the band energy difference. Stability is determined by the structure possessing the lowest energy values, with x_(i)_ denoting the BCC-FCC transition point, x_(ii)_ denoting the BCC-Complex transition point, and x_(iii)_ denoting the Complex-FCC transition point; (**b**) The Fermi Energy difference as defined in equation (4) below; and **(c)** Its associated XRD Patterns.

From Fig. 1(b) it is observed that the FCC phase for the CCFN composition is stabilised in the region of *n* > 6.97 according to the RBA prediction from equation (3). As derived from Eq. (3), the energy difference between two structures (for example FCC-BCC) is dominantly determined by the first contribution coming from spin-polarised band energy difference, 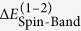
(see dashed and solid plots from Fig. 1(b) where 1: FCC and 2: BCC). The latter expression in turn is derived from Eq. (V) in the Supplementary Information. The spin-polarised band energy is itself a negative quantity representing the attractive contribution to total energy for each structure. Therefore if the FCC-BCC energy difference is negative, then the FCC structure is more stable than the BCC one as shown in Fig. 1. The same conclusion applies to comparison between the relative energy between any third structure and the BCC structure as shown in Figs 2 and 4. More detailed numerical calculations of energy differences for the considered systems are also shown in the Supplementary Table S1.

The FCC stable zone reported by Dominguez *et al*.’s PCA analysis was 8 < VEC < 11^19^; this difference is expected as we have not considered the role of electronic structure in determining the valence electron concentration in different structures. The compositions CCFN-Mn and CCFN-Cu (with *n* = 8 and *n* = 8.8 respectively) lie within the FCC-stable region; this RBA prediction is in agreement with experimental determination of their structures^1^. According to Fig. 1(b), the simple BCC phase would be stabilised within 3.75 < *n* < 6.97. Extension of our RBA analysis to the experimental observation for BCC HEAs containing 4d and 5d BCC transition metals (TMs) previously reported such as WNbMoTa^42^,^43^ with VEC = 5.5, and TiVMnNb^14^ with VEC = 5.25 agrees with the RBA model as both fall within the BCC-stable region.

Below we apply the RBA model to studying the structural-stability competition between simple phases (FCC and BCC) and different complex phases within four CCFN-A_x_ alloys (A = Pd, Al, V, and Ti).

Figure 2(I–IV) shows the results of theoretical and experimental investigation of phase stability of several 5-component CCFN-based alloy systems. In each case the fifth element was chosen to represent a different type of alloying element. In the CCFN-Pd alloys shown in Fig. 2I, palladium is a 4d-transition metal element located on a different row of the periodic table to the other four elements (Cr, Co, Fe and Ni; 3d transition metals). Accordingly, the results from the RBA model comparing the energy difference between FCC and BCC structures in CCFN-Pd alloys, shown in Fig. 2I, are slightly different to those for CCFN alloys displayed in Fig. 1.

When complex structures are included into the RBA analysis as a third type of structure beyond the simple FCC and BCC phases (*cf*. Fig. (2): II, II and IV for consideration of the complex B2, Sigma, and C14 structures; also Fig. (4) for another instance of the complex Sigma structure), Eq. (3) is generalised in the following way: The BCC structure is maintained as the reference system and the RBA calculations are performed by comparing the spin-polarised total energy energies so that Eq. (3) takes the form 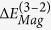 (where 3: Complex phases and 2: BCC phase), as shown in the figure legends below. By using the same reference energy, no fitting parameters are required for determining the stability of this third structure (3) with respect to (1) and (2) and therefore the borders between different phases as a function of *n* may be determined exactly.

The RBA results for energy differences between the simple phases (FCC and BCC) and B2 phase in CCFN-Al alloys (where Al is a sp-metal from outside the transition metal series) are shown in Fig. 2II. Finally, for CCFN-V and CCFN-Ti alloys, where both V and Ti are in the same 3d transition metal series, there is competition between simple and complex phases in term of the Sigma and C14 structures; the RBA model results are shown in Fig. 2III and IV, respectively. The energy differences between complex phases (B2, Sigma, or C14) and the BCC phase calculated using RBA as a function of *n* are shown in Fig. 2I–IV(a). The relationship between the calculated total energy difference between two competing structures (Eq. 3) and the plots of Fermi energy differences, Δ*ε*_F_ shown in Fig. 2I–IV(b) will be explained later in the “*Discussion*” section. The corresponding XRD patterns for CCFN and the four CCFN-A_X_ HEAs are presented in Fig. 2I–IV(c). Peaks observed were attributed to different possible phases: FCC, BCC, B2, Sigma, and C14. In order to compare the theoretical RBA results and the experimental data, the valence electron concentration values *n* calculated within the RBA model for a chosen measured alloy composition are indicated on Fig. 2I–IV(a) and (b) by dashed lines of the same pattern as in the XRD results in Fig. 2I–IV(c).

The FCC-BCC energy difference for CCFN-Pd_x_ HEA obtained using the RBA method shows that the increase of *n* associated with the increasing additions of Pd, stabilises the FCC phase, see Fig. 2I(a). Moreover, the region of stability of single FCC phase contains not only the CCFN and CCFN-Pd_x_ HEA compositions but also it can be extended to *n* = 7. The experimental XRD results for corresponding valence electron concentration values, *n,* confirm the RBA prediction for the CCFN-Pd_x_ HEA since all of the patterns are indexed as the FCC phase. The experimental results also show an increase in the FCC lattice parameter with increasing concentration of Pd, which is in agreement with the most recent fully-relaxed DFT calculations^23^,^35^.

Figure 2II displays the results for RBA analysis for CCFN-Al_x_ where *x* = 0, 0.5, 1.0, and 1.5. The B2 phase is also considered as there is shown to be a high enthalpy of formation of the B2 structure between FeAl, CoAl, NiAl^44^. Moving down Fig. 2II(a),(b), and (c) represents an increase in aluminium content according to corresponding *n* values, and the existence of the B2 phase for CCFN-Al_1.0_ (*n* = 7.2) and CCFN-Al_3.0_ (*n* = 6.81) is confirmed through XRD verification which is also in accordance with the literature^45^,^46^ while CCFN-Al_0.5_ (*n* = 7.67) retains the FCC phase.

In Fig. 2III, for CCFN-V_x_ where *x* = 0, 0.3, 0.8, 1.0, and 2.0, we include consideration of the complex Sigma phase, shown in binary FeCr and FeV phase diagrams^47^. The valence electron concentration value n decreases with increasing V addition. By comparing Fig. 2III(a) and (c), excellent agreement between experiment and stable phases predicted from Δ*E*_Mag_ values as a function of n is found, from single FCC to complex Sigma phase, in accordance with literature^35^.

In Fig. 2IV, for CCFN-Ti_x_ where *x* = 0, 0.4, 0.6, 1.0, and 1.5, the C14 phase is additionally considered as an intermetallic structure related to the CoTi_2_, CrTi_2_, and TiCr_2_ complex phase-forming binary compounds^47^. The inclusion of Ti and the related C14 complex phase into the RBA analysis destabilises the BCC phase much further below *n* = 5.5 than in the previously considered cases of Pd, Al, and V alloying. XRD results show that CCFN-Ti undergoes a transition from the FCC to C14 phase between CCFN-Ti_0.6_ (*n* = 7.70) and CCFN-Ti_1.0_ (*n* = 7.40); the CCFN-Ti_1.0_ (n = 7.40), CCFN-Ti_1.5_ (*n* = 7.09), and CCFN-Ti_2.0_ (*n* = 6.83) phases have been indexed as FCC-C14, C14, and C14-BCC respectively. The structural trend reflecting changes in phase stability between FCC, C14, and BCC will be discussed in the next section.

Generally, the present predictions of phase stability as a function of *n* based on the RBA analysis have been found to give a reasonable match when compared to the outputs from our XRD analysis. Lowering of the value of *n* within families of HEAs causes a change in stability from a FCC single phase to complex phases (B2, Sigma, and C14) back to a BCC single phase. Formation of complex phases is therefore observed close to the transition between FCC and BCC phases, located near *n* = 7 as observed in Fig. 1 for the CCFN alloys. This prediction, based on electronic structure calculations, agrees with Gao’s empirical rules^12^ where complex phase formation is suggested to occur at intermediate *n* values between 6.5 and 7.5.

## Discussion

### Electronic Origin of Phase Stability of Complex Phases in HEA

In order to understand the structural trend from simple to complex phases in CCFN-A_x_ HEAs investigated in the previous section, we begin the analysis of phase stability of CCFN-Al_x_ (where *x* = 0.5, 1.0, and 3.0) within the RBA model by predicting the transition from the FCC to the B2 phase. It is worth emphasising again that the RBA has been successfully applied to the investigation of structural trends in intermetallic compounds and complex Hume-Rothery phases in transitional metal aluminides known as *spd electron phases*^24^,^26^. Here the hybridisation effect between sp-valence electrons of Al with the d-orbitals of transition metals (TM) plays a crucial role in structural stability trends and their physical properties. In the case of CCFN-Al_x_ HEAs, a similar effect can be seen from the construction of frozen-potential approximation for Al and TM atoms (Cr, Co, Fe, Ni) to the electronic structure calculations within the RBA model for different SQS structures. It is well-known from both experimental and DFT data that among the B2 compounds formed between Al and TMs, the B2-AlNi phase has the strongest negative enthalpy of formation^44^. The B2 phase can be constructed, therefore, from an ordered structure with four Al, Ni, Fe, Co atoms where Al-Ni pairs are dominant at nearest neighbour distances in the BCC-like structure.

Figure 2IIa shows the B2-BCC structural energy difference calculated from the RBA model plotted together with the FCC-BCC difference as a function of *n*. It is found from the RBA calculations that competition between the FCC and B2 phases starts at valence electron concentration, *n* ≈ 8. Comparison with the present experimental data for CCFN-Al_0.5_ (*n* = 7.67) where FCC peaks are indicated in XRD (Fig. IIc), shows that the RBA model may overestimate the stability of the B2 phase.

However, in comparison with a new HEA composition, AlCoCrFeNi_2.1_, where the corresponding valence electron concentration is *n* = 7.70, the experimental observation^48^ of an FCC/B2 dual-phase constitution. Furthermore, experimental evidence of B2 presence has also been reported in CCFN-Al_0.75_, CCFN-Al_1_, and CCFN-Al_1.5_ with *n* values ranging from 6.57 to 7.41^49^. These observations not only validate our theoretical prediction but also demonstrate that the formation of B2 phase is strongly correlated with the short-range chemical order between Al and the excess composition of Ni transition metals in the HEAs, suggesting possible effects of the processing method and cooling rate, as noted by Dahlborg *et al*.^14^.

The phase stability trends involving the FCC phase and intermetallic phase C14 for the CCFN-Ti_x_ compositions in Fig. 2IV also deserve further analysis. From Table 1, we observe a change in experimentally determined phase stabilities from CCFN-Ti_1.0_, CCFN-Ti_1.5_, and CCFN-Ti_2.0_ as a mixture of FCC-C14, to C14, and finally a BCC-C14 mixture, respectively. By comparing the theoretical results displayed between Figs 1 and 2IV, it is found that the CCFN-Ti_1.0_ composition with *n* = 7.40 is located within the CCFN FCC stable region while the CCFN-Ti_2.0_ composition with *n* = 6.83 is below the CCFN FCC-BCC nodal point at *n* = 7.26. The CCFN-Ti_0.5_ composition with *n* = 7.09 lies closest to the FCC-BCC nodal point where Δ*E*_Mag_ = 0, suggesting that complex phases form at these points, which is in line with the analysis performed by Dominguez *et al*.^19^ where the stability was found to range from the simple FCC phase (high VEC values), to complex phases (medium VEC values), to the simple BCC phase (low VEC values).

### Relative Structural Stability and their Fermi Energy Difference, Δ*ε*_F_

The relative structural stability of two phases within the RBA model is defined by comparing the spin-polarised band-structure energy difference in equation (3) as a function of the valence electron concentration, *n*, determined from equation (2). The origin of the structural stability within the RBA model can be further analysed in terms of the change in the Fermi energy^24^,^25^. This can be defined as the first derivative of the band energy difference of two competing structures with respect to the change in *n*:


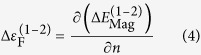


From equation (4) it follows that an extremum of the energy difference, Δ*E*_*Mag*_ occurs for the number of electrons at which the two Fermi energies are equal, i.e. 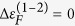. This condition is important because it corresponds to points where the second phase becomes more stable in comparison to the first phase^24^,^25^. For the CCFN case shown in Fig. 1, 
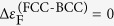
 at *n* = 5.5 where 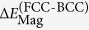 is a maximum and the BCC phase exhibits the largest stability with respect to the FCC phase, see Fig. 1(b) and (c). Applying the criterion (4) to CCFN-Pd_x_, the maximum difference between FCC and BCC phase is predicted at *n* = 9.4, where Δ*ε*_F_ = 0, as can be seen in Fig. 2I(b). For that value of *n* the FCC phase is more stable in comparison with the BCC phase.

Figure 2II(b) shows Δ*ε*_F_ plots calculated from the energy difference Δ*E*_Mag_ for both the FCC and B2 phases in CCFN-Al_x_ HEA in reference to the BCC phase. The maximum difference Δ*E*_Mag_ between the FCC-BCC phases is at *n* = 7.8, which in turn corresponds to Δ*ε*_F_ = 0, but the former is not the most stable structure since Δ*E*_Mag_ of the B2-BCC phases is lower than that of FCC-BCC phases at this value, as shown in Fig. 2II(a). A plot of Δ*ε*_F_ as a function of *n* for B2-BCC phases shows that there are two zero values at *n* = 6.4 and 7.4 where the B2 phase could be the most stable. The differentiation between these phases with XRD as used in the present study cannot be certain due to the low intensity of the peaks, but B2 formation appears to begin from CCFN-Al_0.5_ (*n* = 7.67) and continues in CCFN-Al_1.0_ (*n* = 7.2) and CCFN-Al_1.5_ (*n* = 6.81). This agrees with the determined value of *n* = 7.4, where the Fermi energy difference between B2 and BCC is equal to zero. It is worth highlighting that by looking at the general competition between FCC, B2, and BCC phases the criterion Δ*ε*_F_ = 0 strongly supports the experimental observation of dual FCC/B2 phases observed in AlCoCrFeNi_2.1_ at *n* = 7.7, which is located between the FCC stable phase (*n* = 7.8) and the B2 stable phase (*n* = 7.4).

For CCFN-V from Fig. 2III(b) it appears that for the maximum stability of the Sigma phase with respect to the BCC phase, two zero values for Δ*ε*_F_ at *n* = 7.65 and *n* = 6.65 are observed. From XRD results it is evident that the Sigma phase is dominant between CCFN-V_1.0_ and CCFN-V_2.0_ (*n* = 7.6 and *n* = 7.16 respectively), and CoFN-V_1.5_ and CoFN-V_2.0_ (*n* = 7.67 and *n* = 7.4, respectively). This is also close to the predicted value of the most stable Sigma at *n* = 7.65.

According to Fig. 2IV(b) for the CCFN-Ti family, the predicted stationary point of Δ*ε*_*F*_ = 0 from the FCC-BCC plot, corresponding to the most stable FCC point, lies at *n* = 9.0. The stationary point for the C14-BCC plot is located at *n* = 6.4 where the C14 phase is most stable. The C14 phase is not observed as a stable structure of CCFN-Ti_0.4_ at *n* = 7.85 and CCFN-Ti_0.6_ at *n* = 7.7 because from Fig. 2IV(a) Δ*E*_Mag_ of the FCC-BCC plot is lower than the C14-BCC one for *n* ≥ 7.69. It is possible that C14 phases may exist in small quantities and further precipitation is being supressed by the high cooling rate as a result of the synthesis method used in this work.

Thus, beside the case of CCFN-Pd_x_ HEA, where the FCC phase is the most stable for all *n* values (see Fig. 2I(b)), the above analysis using the criterion Δ*ε*_F_ = 0 allows compositions to be predicted in CCFN-Al_x_, CCFN-V_x_, CCFN-Ti_x_ HEAs where complex phases are stable and these alloy families therefore possesses the ability to develop microstructures consisting of simple/complex phase combinations.

### RBA versus Experimental observation of HEA Phase Stability as a Function of n

**Figure 3:**
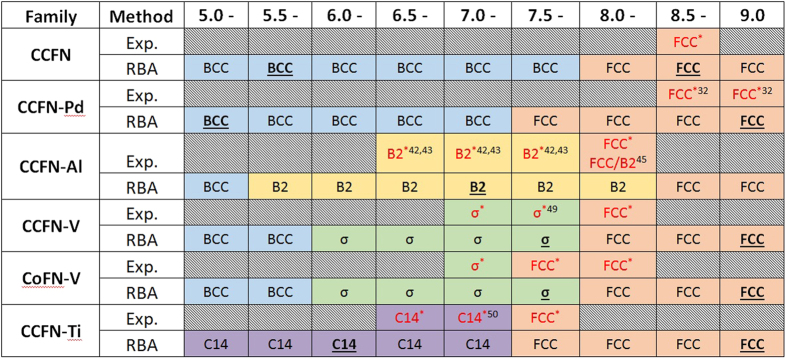
Phases present via XRD (Red) as a function of regions of valence electron concentration and predicted via Δ*E*_*Mag*_ (Black) and Δ*ε*_*F*_ = 0 (**Bold**,**Underlined**) as a function of regions *n*. Both VEC and *n* are considered equivalent here, showing good interchangeability between both. While Δ*E*_*Mag*_ predicts the relative stability of different phases (*c.f.* equation (3)), Δ*ε*_*F*_ = 0 represents the critical point at which the considered phase is the most stable one (*c.f.* equation (4)). *Indicates experiments performed in this work, while a superscript number indicates that the composition is found in the corresponding reference from the literature possessing the indicated structure.

Figure 3 shows a comparison of the experimentally-determined phase present and quantum-mechanical RBA-determined stable phase for each HEA family with different values of *n*. The first row for each HEA family represents the former as determined from Δ*E*_*Mag*_ while the indicated (bolded, underlined) regions show the points at which Δ*ε*_*F*_ = 0 where the indicated phase is the most stable. It is shown that the agreement between experimental and RBA results is very satisfactory. In the case of CCFN-Al_x_, the present experimental XRD results of the BCC phase in the range of Al concentration between 0.5 (*n* = 7.67) and 1.0 (*n* = 7.2) show that these alloys are strongly disordered, whereas the Al-Ni short range chemical-order alloys present within the RBA model are more in favour of the B2 phase. The experimental observation of dual FCC/B2 phases in the Ni-rich HEA of AlCoCrFeNi_2.1_ (*n* = 7.70) has confirmed the validity of the RBA prediction. In general, it is observed that the simple FCC phase is present at *n* > 8, complex phases are present between 6 < *n* < 8, and the simple BCC phase at *n* < 6. This variation in phase stability as a function of *n* supports the simple two dimensional plots presented by Dominguez *et al*.^14^ and Guo *et al*.^17^. In particular, the empirical VEC parameter used in all previous studies has a strong relationship with the quantum-mechanical value *n* from electronic-structure calculations.

It is apparent from Fig. 2 and summarised in Fig. 3 that performed in conjunction, analysis of Δ*E*_Mag_ and Δ*ε*_F_ results allow for deeper understanding of phase stabilities, although for rough predictions Δ*E*_Mag_ alone is enough to give relatively accurate results for the compositions tested in this work. A comparison of Δ*E*_Mag_ and Δ*ε*_F_ with *n* values for the tested compositional families show that in terms of phase stability, *n* at which the simple FCC phase transition is located, is dependent on the chemical bonding nature of the alloying element to CCFN.

The results of the RBA analysis suggest that empirical VEC values have a strong connection to the values of *n* calculated from electronic spin-polarised DOS (Eq. 2) and that the accuracy of predictions in alloy design can be improved as long as electronic structure effects at the quantum scale are accounted for. In light of the dependency on the electronic structure, the increased accuracy with which HEA complex phases may be determined is not surprising. As Miedema’s empirical rule for the enthalpy of mixing, ∆H, has been found to be inconsistent with quantum mechanical principles^50^,^51^, the ability of the two-dimensional plot shown by Dominguez *et al*.^19^ to distinguish between components may arise from the deviation in the ratio between the enthalpy of mixing and the difference in the number of valence electrons squared from Miedema’s model. The latter quantity Δ*H*/(Δ*n*)^2^ has been shown^51^ to start deviating from theoretical predictions between 4 < *n* < 7, which intersects with what are regarded as zones of complex phase presence in HEAs.

These factors may be accounted for by utilising the RBA technique presented in this paper. As shown in Figs 2 and 3, Δ*E*_Mag_ values may be used to approximate, to a good degree of accuracy, the phases present in any particular stoichiometric composition within a preselected CCFN family (here family refers to all elemental alloying components comprising the composition), as a function of *n*.

### Structural Stability of the New HEAs: CoFeNi-V_x_

The above hypothesis is tested through the removal of Cr from CCFN-V_x_ to form CoFeNi-V_x_ (here denoted as CoFN-V_x_) alloys and the analysis of phase stabilities utilising the RBA method as a function of changes in vanadium addition. To achieve this, we modify the CCFN FCC-BCC RBA analysis to include consideration of the Sigma phase due to strong enthalpies of mixing of FeCr and FeV for the Sigma phase, as in the case of CCFN-V_x_. No explicit consideration is necessary as Co, Cr, Fe, Ni, and V are located on the same row of the periodic table.

**Figure 4:**
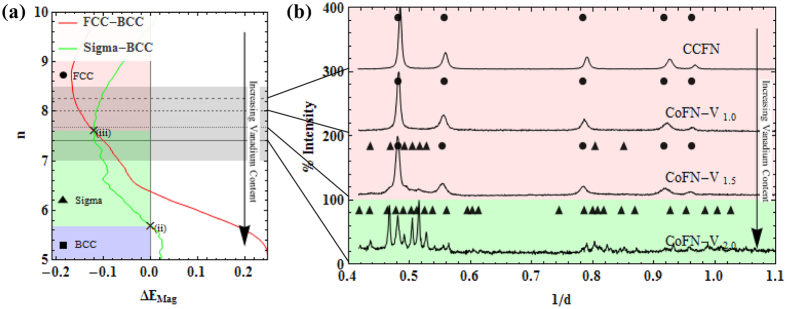
Transition of FCC to Sigma phase stability in CoFN-V. (**a**) As a function of increasing Vanadium content represented by *n*, and (**b**) Its associated XRD Patterns.

Figure 4 indicates the analysis of the CoFN-V_x_ composition, in terms of *n*. The RBA analysis described in Fig. 2(III) for the relative structural energy between FCC-BCC and FCC-Sigma phases in CCFN-V_x_ is adapted for investigation of the new HEAs. Removal of Cr has the effect of shifting *n* values to higher regions, with the effect of destabilising the Sigma structure. This shift in *n* to the region of FCC stability may be attributed to the Fermi surface nesting in HEAs with or without Cr which can stabilise a complex phase^52^. For equiatomic CoFN-V_1.0_ at *n* = 8 we observe from Fig. 4(a) that the FCC phase is stabilised as compared to the presence of Sigma phase previously considered at equiatomic CCFN-V_1.0_ with *n* = 7.6. In Fig. 4(b) experimental XRD patterns verify the prediction of CoFN-V_1.0_ and subsequent compositions, CoFN-V_1.5_ (FCC phase at *n* = 7.67) and CoFN-V_2.0_ (Sigma phase at *n* = 7.40) predicted within the RBA model, indicating that the VEC values in Table 1 are in good agreement with *n* and that the RBA method therefore can be used as a valid tool for phase prediction, by taking into consideration the chemical bonding of the alloying species.

The power of the RBA model when used in conjunction with *n* in predicting complex phase formation in HEAs makes it suitable for the design of new HEA compositions. The relative phase stability as a function of *n* may be analysed for equiatomic compositions of designated multi-component alloys with four or more elements. As formation of the complex phase is identified to occur around the nodal points of FCC-BCC energy difference where Δ*E*_Mag_ = 0 (*c.f*. Fig. 2), the generalisation of the RBA approach would require a starting alloy composition that is a known simple phase (FCC/BCC) such as CCFN (FCC), CCFN-Pd (FCC), CCFN-Mn (FCC), WNbMoTa (BCC), or TiVMnNb (BCC), which is then modified by changing its stoichiometry, or by further alloying additions to the composition. It is preferable for the initial structure to be FCC-type as it is less complicated to obtain the self-consistent charge density from a close-packed structure as an input frozen potential for the RBA, as compared to a more complex structure. The secondary phase chosen for consideration will depend on the alloying additions and the enthalpies of mixing of the phases, which may either be analysed directly or obtained from literature.

This alloy development strategy could become a platform to analyse specific compositions of interest and explore the effects of further additions on that particular system along the entire compositional range. Furthermore, phases of particular interest, such as the Laves phase for high-temperature structural materials, may be used as a starting point. The vast choice of combinations from within the periodic table could further diversify the compositions and provide strategies to optimise the mechanical properties, such as by changing the phase fraction and composition, or by including specific elements that individually would be possibly expected to improve chemical properties such as corrosion resistance (Cr, Mo), radiation shielding alloys (W), or biocompatibility (Pd) etc.

## Conclusions

In this paper we have applied the RBA approach generalised for magnetic multi-component HEA systems and determined by Eq. (3) that gives a full description of the influence of electronic structure effects on phase formation in HEAs, and compared the predictions to experimental results. Our main findings are as follows:

- The RBA model proves successful in predicting phases present in multicomponent HEAs as a result of macro alloying of the CCFN composition. The formation of complex phases is found to coincide with the transition between FCC and BCC, being the lower energy structures at intermediate values of the valence electron concentration, *n* determined from the integration of spin-polarised DOS computed for magnetic HEAs. The model however, does not take into account the effects of atomic rearrangement on the phase stability resulting from thermomechanical processing (due to the near-ideal nature of the simple phase HEAs). Therefore in follow-up studies it may be interesting to adapt the RBA model to be used in combination with other methods (such as Monte-Carlo simulations^43^) to study the kinetic stability of HEAs.
- Values of *n*, for which complex phases are found, were predicted with more precision for specific alloy systems, and predictions were validated experimentally: the Sigma phase for CCFN-V_x_ (*n* < 7.6), B2 for CCFN-Al_x_ (*n* < 8.1), and C14 phase for CCFN-Ti_x_ (*n* < 7.7).
- The RBA scheme is successful in predicting the presence of complex Sigma phase in the previously unreported CoFN-V_x_ composition (*n* ≤ 7.6) and allows the prediction of other complex intermetallic phases in various new alloy compositions as a function of *n*. A design strategy based on this approach would be that through a careful selection of elements and/or utilising a known HEA composition with high *n* as a ‘base’ phase (FCC), it would be possible to include elements of interest for purposes such as corrosion resistance, biocompatibility, and high-temperature resistance, while mechanical properties could be influenced by changing the phase fraction and composition.

## Methods

### Sample Preparation

Samples were prepared by arc-melting elements of at least 99.9% purity in a water-cooled copper hearth in an argon atmosphere. The ingots were re-melted three times for good mixing and sectioned in half to be visually inspected for segregation. Samples with segregation or unmelted constituents were re-melted a further three times until good mixing was achieved. Following this the samples were then suction-cast as 3 mm diameter rods in a water-cooled copper mould.

### X-Ray Diffraction (XRD) Characterisation

Transmission XRD experiments of powder produced by rasping samples in the as-cast condition were conducted on a STOE Stadi diffractometer utilising a Mo k-α monochromated source. This method was chosen to allow characterisation of samples that proved to be brittle. Through comparison of experimental data with equivalent literature data it was found that similar phases present in the XRD pattern despite the lower resolution of the radiation source. All samples were run from 17°–50° 2ϑ with a step size of 0.02 for four hours and the XRD patterns were Rietveld refined using reference instrument diffraction profiles. Figures showing the experimental patterns together with the simulated profiles and residuals may be viewed in the supplementary information.

All XRD values are presented as a function of the percentage intensity and reciprocal lattice, *d*^−1^. Characterisation of the XRD results was performed utilising indexed patterns from the PDF4+ database as a guide; following which a Rietveld refinement on the lattice parameters and structure was performed on the data using the GSAS software package.

It should be recognised that, while commonly used for characterisation of the structure of metals and alloys, standard XRD may not detect lower intensity peaks associated with smaller phase fractions due to low resolution or poor signal-to-noise ratios, and also may not discriminate precisely between such effects as depth profile information and compositional variation from segregation, for example. However, for the current goal it is a suitable characterisation method as the RBA technique is itself a DFT-based electronic structure method suitable for predictions in situations of incomplete knowledge, and a complete experimental investigation of structure and properties would naturally follow.

### Computational details

The frozen potential calculations have been performed using full-potential linear muffin-tin orbital LMTO code^28^ allowing the self-consistent potentials DFT calculations to be carried out for RBA investigation. For each considered CCFN-based alloy both non-magnetic and spin-polarised electronic densities of states were obtained in order to predict the effective Stoner parameter, and the band energies and average magnetic moments as functions of the number of valence electrons per atom respectively. The investigated structures were assumed to be either fully disordered for the simple FCC and BCC phases or partially disordered for the complex B2, C14, and Sigma phases, where the chosen elements were preferentially occupying one of the sub-lattices. These structures were generated by using the special-quasi random structures method^29^,^30^. The Wigner-Seitz (WS) spheres in FCC, BCC, B2, and Sigma phases were assumed to be equal for different atomic species whereas in the C14 phase the WS spheres occupied by Cr atoms were assumed to be larger than others. Further details on the RBA may be found in the attached supplementary information.

## Additional Information

**How to cite this article**: Leong, Z. *et al*. The Effect of Electronic Structure on the Phases Present in High Entropy Alloys. *Sci. Rep.* **7**, 39803; doi: 10.1038/srep39803 (2017).

**Publisher's note:** Springer Nature remains neutral with regard to jurisdictional claims in published maps and institutional affiliations.

## Supplementary Material

Supplementary Information

## Figures and Tables

**Table 1:** High Entropy Alloy compositions with their respective nominal compositions, indexed phases and lattice constants, and VEC values.

Composition	Nominal Composition	Indexed Phase	Lattice Parameter (Å) +/− 0.01 Å	VEC	Reference
CCFN	CoCrFeNi	FCC	*a* = 3.56	8.25	9,13, 14, 15,17,35
CCFN-Pd_0.5_	(CoCrFeNi)_0.89_Pd_0.11_	FCC	*a* = 3.62	8.44	14,35
CCFN-Pd_1.0_	(CoCrFeNi)_0.80_Pd_0.20_	FCC	*a* = 3.66	8.60	14,35
CCFN-Pd_1.5_	(CoCrFeNi)_0.73_Pd_0.27_	FCC	*a* = 3.71	8.73	14
CCFN-Al_0.5_	(CoCrFeNi)_0.89_Al_0.11_	FCC	*a* = 3.60	7.67	45,46
		BCC	*a* = 2.87		
CCFN-Al_1.0_	(CoCrFeNi)_0.80_Al_0.20_	BCC	*a* = 2.88	7.20	45,46
CCFN-Al_1.5_	(CoCrFeNi)_0.73_Al_0.27_	BCC	*a* = 2.88	6.81	45,46
		B2	*a* = 2.82		
CCFN-Al_3.0_	(CoCrFeNi)_0.57_Al_0.43_	B2	*a* = 2.89	6.00	This Work*
CCFN-V_0.3_	(CoCrFeNi)_0.93_Al_0.07_	FCC	*a* = 3.58	8.02	This Work*
CCFN-V_0.7_	(CoCrFeNi)_0.85_V_0.15_	FCC	*a* = 3.59	7.77	This Work*
		Sigma	*a* = 8.78, *c* = 4.60		
CCFN-V_1.0_	(CoCrFeNi)_0.80_V_0.20_	FCC	*a* = 3.61	7.60	53
		Sigma	*a* = 8.79, *c* = 4.58		
CCFN-V_2.0_	(CoCrFeNi)_0.67_V_0.33_	Sigma	*a* = 8.87, *c* = 4.59	7.17	This Work*
CoFN-V_1.0_	(CoFeNi)_0.75_V_0.25_	FCC	*a* = 3.59	8.00	This Work*
CoFN-V_1.5_	(CoFeNi)_0.67_V_0.33_	FCC	*a* = 3.61	7.67	This Work*
CoFN-V_2.0_	(CoFeNi)_0.60_V_0.40_	Sigma	*a* = 9.04, *c* = 4.68	7.40	This Work*
CCFN-Ti_0.4_	(CoCrFeNi)_0.91_Ti_0.09_	FCC	*a* = 3.59	7.86	This Work*
CCFN-Ti_0.6_	(CoCrFeNi)_0.87_Ti_0.13_	FCC	*a* = 3.61	7.70	This Work*
CCFN-Ti_1.0_	(CoCrFeNi)_0.80_TI_0.20_	C14	*a* = 4.79, *c* = 7.76	7.40	54
		FCC	*a* = 3.64		
CCFN-Ti_1.5_	(CoCrFeNi)_0.73_TI_0.27_	C14	*a* = 4.77, c = 7.74	7.09	This Work*
CCFN-Ti_2.0_	(CoCrFeNi)_0.67_Ti_0.33_	C14	*a* = 4.81, *c* = 7.82	6.83	This Work*
		BCC	*a* = 2.98		

^*^These new stoichiometric compositions have been selected to extend the literature data on the CCFN-A (A = Pd, V, Al, and Ti) compositional families selected for this work.
