# Supplementary material for: The Effect of Electronic Structure on the Phases Present in High Entropy Alloys
Source: Sci Rep. 2017 Jan 6;7:39803. doi: 10.1038/srep39803 (PMC5216361; doi:10.1038/srep39803)
Supplement: Supplementary Information [file srep39803-s1.pdf]

The Effect of Electronic Structure on the Phases Present in High Entropy Alloys

Zhaoyuan Leong[1*], Jan S. Wróbel[2,3], Sergei L. Dudarev[2], Russell Goodall[1], Iain Todd[1], Duc Nguyen-Manh[2**]

Supplementary Information

Rigid Band model

The structural stability of simple FCC and BCC phases, and all other complex phases observed in multi-component alloys can be investigated by using the frozen potential approximation (FPA) to density functional theory (DFT). Within the first order approximation the energy difference between any two non-magnetic structures is given by the difference between their band energies at a fixed equilibrium volume, V_0 [35,36,49]:

$$\Delta E^{(1-2)}_{Non-Mag} \approx \Delta E^{(1-2)}_{Band} = \left[2\int_{-\infty}^{\varepsilon_F^{(1)}} \varepsilon D^{(1)}(\varepsilon)d\varepsilon - 2\int_{-\infty}^{\varepsilon_F^{(2)}} \varepsilon D^{(2)}(\varepsilon)d\varepsilon \right]_{\Delta V_0=0} \quad \text{(I)}$$

where $D^{(1)}(\varepsilon)$ and $D^{(2)}(\varepsilon)$ are the densities of states (DOS) per atom with a factor of 2 for a spin to energy ε and $\varepsilon_F^{(1)}$ and $\varepsilon_F^{(2)}$ are the Fermi energies for structure 1 and 2, respectively. They are determined from the number of valence electrons per atom:

$$n = 2\int_{-\infty}^{\varepsilon_F^{(1)}} D^{(1)}(\varepsilon)d\varepsilon = 2\int_{-\infty}^{\varepsilon_F^{(2)}} D^{(2)}(\varepsilon)d\varepsilon \quad \text{(II)}$$

Frozen potentials for the different atomic species are determined from self-consistent calculations, normally for structures with a small unit cell. They are then transferred into other structures with the same alloy composition and the same equilibrium volume. The difference between the band energies $\Delta E^{(1-2)}_{Band}$ is calculated under the constraint that the potential within the Wigner-Seitz sphere remains unchanged (frozen) when going from one structure type to another. Since the energy difference in the first order frozen potential approximation is computed assuming fixed atomic volumes, this approximation is correct for structures with relatively similar equilibrium atomic volumes like for example FCC and BCC phases, which can be related to each other through the Bain transformation.

Assuming that alloying changes the number of valence electrons per atom but the electronic DOS of different phases remain rigid (the rigid band approximation), the variation of the energy difference as a function of the number of electrons can be evaluated by comparing the band energies, see equation (I)[19,20,50].

Within the present work, the RBA model has been generalised for the case of magnetic materials like CCFN-based alloys using the Stoner model of magnetism[23–27]. In transition metal alloys the latter one introduces magnetism by introducing local exchange fields within the band energy concept. In particular, electrons with spins up and down have different on-site energies depending on whether their spin is parallel or anti-parallel to the local magnetic moment. The effective on-site energy ε_i^{σ} is given by

$$\varepsilon_i^{\sigma} = \varepsilon_i \pm \frac{1}{2} I_i m_i \quad \text{(III)}$$

where ε_i is the single-particle non-magnetic on-site energy, $m_i = n_i^{\downarrow} - n_i^{\uparrow}$, is the difference between electron occupancies of site i by spin-down and spin-up electrons with a spin index σ. The Stoner exchange parameter I_i refers to the exchange splitting of on-site energies of electrons with spin up and spin down due to the local magnetic moment m_i [23,24,26,27,30].

From equation (III), the energy difference between two magnetic structures, following the Stoner model of magnetism (Stoner Model I), corrected for the double counting contribution, and normalised by the number of atoms, N in both structures can be given by:

$$\Delta E_{Mag}^{(1-2)} = \Delta E_{Spin-Band}^{(1-2)} + \frac{1}{4N}\left[\sum_i I_i^{(1)} m_i^{2(1)} - \sum_i I_i^{(2)} m_i^{2(2)}\right] \quad \text{(IV)}$$

where the first term is the spin-polarised band energy difference between any two magnetic structures at a fixed equilibrium volume V_0 (defined below in equation (V)), the second term is the double-counting contribution arising from magnetic interactions[23–25], and N is the number of atoms in both considered structures. $\Delta E_{Spin-Band}^{(1-2)}$ is defined as:

$$\Delta E_{Spin-Band}^{(1-2)} = \left[\int_{-\infty}^{\varepsilon_F^{(1)}} \left(\varepsilon D^{(1)\uparrow}(\varepsilon) + \varepsilon D^{(1)\downarrow}(\varepsilon)\right) d\varepsilon - \int_{-\infty}^{\varepsilon_F^{(2)}} \left(\varepsilon D^{(2)\uparrow}(\varepsilon) + \varepsilon D^{(2)\downarrow}(\varepsilon)\right) d\varepsilon\right]_{\Delta V_0 = 0} \quad \text{(V)}$$

where $D^{(1)\uparrow}(\varepsilon)$ and $D^{(2)\uparrow}(\varepsilon)$ are the spin-polarised total DOS per atom for electrons with spin-up and $D^{(1)\downarrow}(\varepsilon)$ and $D^{(2)\downarrow}(\varepsilon)$ are the spin-polarised total DOS per atom for electrons with spin-down. The Stoner parameters in equation (IV) are assumed to be constant for each type of atoms[51]**.** However, application of equation (IV) requires knowledge of the magnetic moments of all atoms as a function of *n* in the system, of which is difficult to be validated experimentally. Therefore, we use an effective Stoner parameter I_{eff} which refers to the exchange splitting of the on-site energies of electrons with spin-up and spin-down due to the average atomic magnetic moment m_{av}, defined as:

$$I_{eff}\, m_{av} = \varepsilon_F^{\downarrow} - \varepsilon_F^{\uparrow} \quad \text{(VI)}$$

where $\varepsilon_F^{\uparrow}$ and $\varepsilon_F^{\downarrow}$ are the Fermi energies for electrons with spin-up and spin-down, respectively, defined as energies of the non-magnetic total DOS, $D(\varepsilon)$, which are the number of electrons per atom with spin-up and spin-down, obtained from the spin-polarised total DOS. The average magnetic moment of the entire simulation cell from the non-magnetic total DOS is equal to the one obtained from the spin-polarised total DOS and is given by:

$$m_{av} = \int_{-\infty}^{\varepsilon_F} D^{\downarrow}(\varepsilon) d\varepsilon - \int_{-\infty}^{\varepsilon_F} D^{\uparrow}(\varepsilon) d\varepsilon = \int_{\varepsilon_F^{\uparrow}}^{\varepsilon_F^{\downarrow}} D(\varepsilon) d\varepsilon \quad \text{(VII)}$$

By using the effective Stoner parameter and the average magnetic moment the energy difference between any two magnetic structures in the effective Stoner Model II can now be written as:

$$\Delta E_{Mag}^{(1-2)} = \Delta E_{Spin-Band}^{(1-2)} + \frac{1}{4}\left[I_{eff}^{(1)} m_{av}^{2\,(1)} - I_{eff}^{(2)} m_{av}^{2\,(2)}\right] \quad \text{(VIII)}$$

Both Stoner Model I and effective Stoner Model II can be justified by the comparison of energy differences with those obtained using the LMTO code. As shown in Table SI the energy differences between the considered magnetic structures and the BCC structures with the same composition calculated using both equation (IV) and (VIII) are in quantitative agreement with each other and in line with the results calculated using the LMTO code. The only exception is that the C14 structure of $Co_9Cr_{12}Fe_9Ni_6$ alloy which is less stable than the BCC phase when equation (VIII) is applied and it is more stable than the BCC phase by using equation (IV) and the LMTO code. However, all the considered methods predict that the FCC phase is the most stable one.

Supplementary Table SI. Average magnetic moments obtained using equation (VII) (m_{av}) and energy differences (ΔE_{mag}) between the considered magnetic structures and the BCC structures with the same composition calculated using equations (IV) and (VIII), compared with the results calculated using the LMTO code. I_{eff} is the effective Stoner parameters calculated using equation (VI). Energies, magnetic moments and effective Stoner parameters are given in eV , μ_β per atom and eV / μ_β, respectively. The valence electron concentration of considered structures is indicated by *n*.

	Structure	ΔE_{Mag} (equation (IV))	ΔE_{Mag} (equation (VIII))	ΔE_{Mag} (LMTO)	m_{av} (equation (VII))	m_{av} (LMTO)	I_{eff} (equation (VI))
**CCFN $Co_8Cr_8Fe_8Ni_8$ (*n* = 8.25)**	FCC	-0.136	-0.099	-0.083	0.372	0.372	1.295
	BCC				0.421	0.421	1.302
**CCFN-Al $Co_8Fe_8Ni_8Al_8$ (*n* = 7.5)**	FCC	-0.043	-0.020	-0.001	0.939	0.930	3.940
	BCC				1.011	0.990	4.392
	B2	-0.065	-0.102	-0.072	0.948	0.948	4.124
**CCFN-Pd $Co_6Cr_7Fe_7Ni_6Pd_6$ (*n* = 8.5)**	FCC	-0.139	-0.070	-0.064	0.881	0.864	3.631
	BCC				0.913	0.915	3.746
**CCFN-V $Co_9Cr_{12}Fe_9Ni_6$ (*n* = 8.133)**	FCC	-0.147	-0.123	-0.073	0.350	0.344	1.228
	BCC				0.415	0.422	1.205
	Sigma	-0.151	-0.120	-0.069	0.312	0.317	0.993
**CCFN-Ti $Co_9Cr_{12}Fe_9Ni_6$ (*n* = 7.917)**	FCC	-0.063	-0.027	-0.021	0.687	0.171	0.987
	BCC				0.691	0.172	0.831
	C14	-0.018	0.022	-0.013	0.817	0.819	0.909

XRD Data Rietveld refined peaks

The following figures show the zoomed-in plots of the XRD data from the main text. The raw experimental data is shown as the orange lines, whilst the Rietveld simulated lines are shown as a black line. Rietveld refinement was performed using the input information of the phases as determined from existing data from literature as shown in Table 1 of the main text; the lattice parameters that are listed in Table 1 result from the Rietveld refinement performed. The brown line indicates the residuals that arise from the difference between the experimental and simulated patterns.

For all compositions, the chi-square, χ^2 values of the refinement are all noted to be lower than 4. A visual comparison of the experimental pattern to the simulated patterns shows that all peaks of more than 5% percentage intensity have been indexed. Some peaks in the CCFN-$Ti_{1.0}$ composition was not fully indexed due to the complexity of the complex phase. Some low intensity peaks may also be present in the CCFN-$Al_{1.0}$ compositions that are not indexed due to the low signal but are expected to not be present in significant amount. In general, the results show that the majority structure of the HEA systems presented here have been successfully indexed. The refined phases and lattice parameters are as presented in Table 1 of the main text.

CCFN and CCFN-Pd_x systems

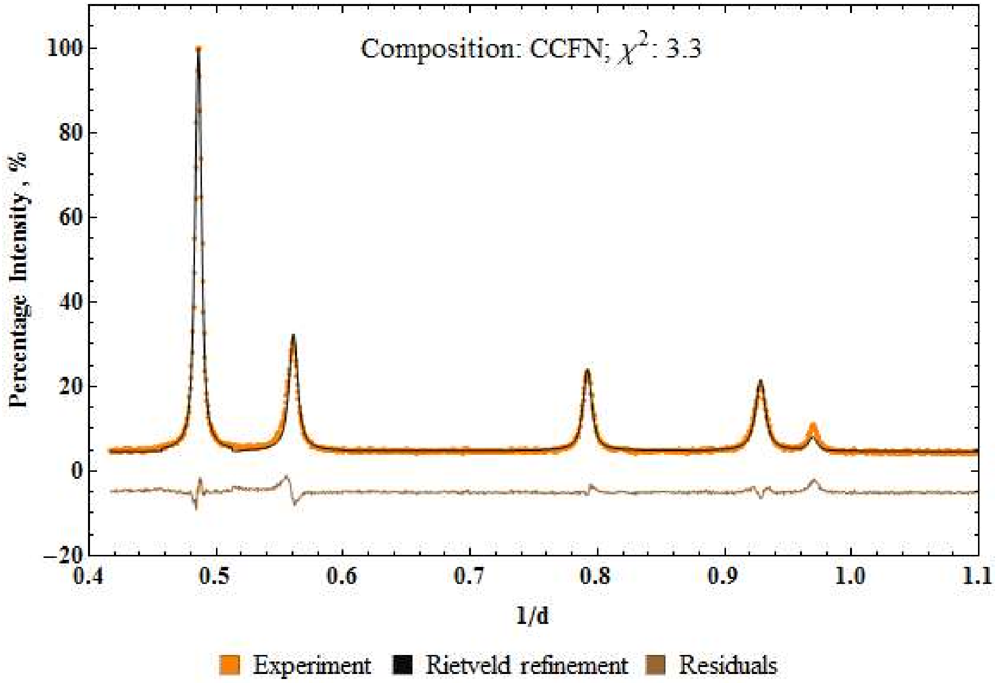

Supplementary Figure S1. CCFN experimental pattern (Orange), Rietveld simulated pattern (Black), and residuals (Brown).

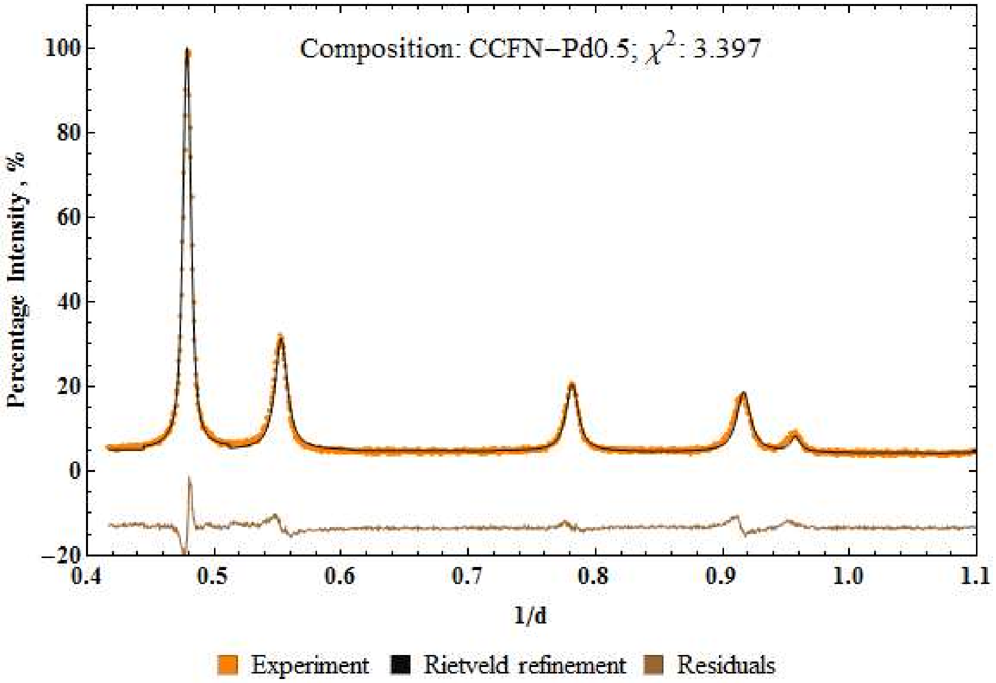

Supplementary Figure S2. CCFN-$Pd_{0.5}$ experimental pattern (Orange), Rietveld simulated pattern (Black), and residuals (Brown).

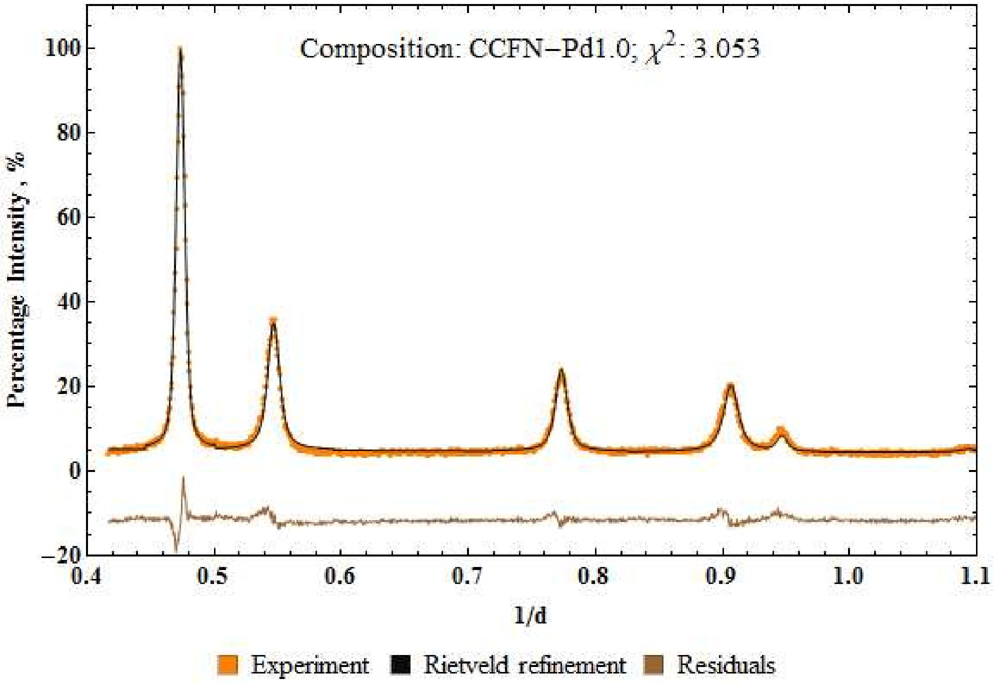

Supplementary Figure S3. CCFN-$Pd_{1.0}$ experimental pattern (Orange), Rietveld simulated pattern (Black), and residuals (Brown).

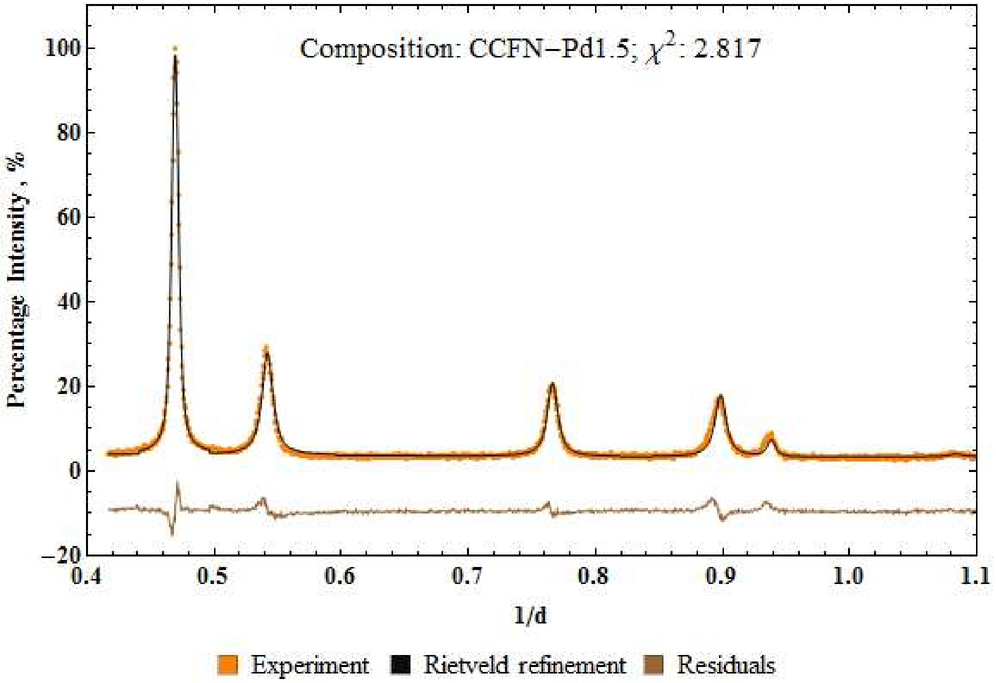

Supplementary Figure S4. CCFN-$Pd_{1.5}$ experimental pattern (Orange), Rietveld simulated pattern (Black), and residuals (Brown).

CCFN-Al$_x$ systems

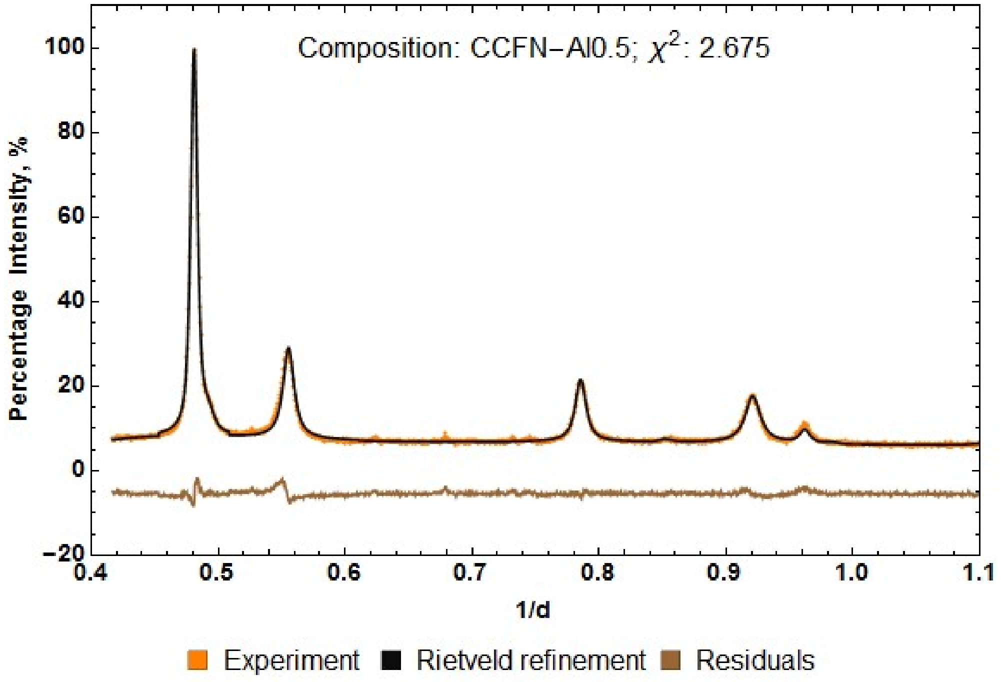

Supplementary Figure S5. CCFN-$Al_{0.5}$ experimental pattern (Orange), Rietveld simulated pattern (Black), and residuals (Brown).

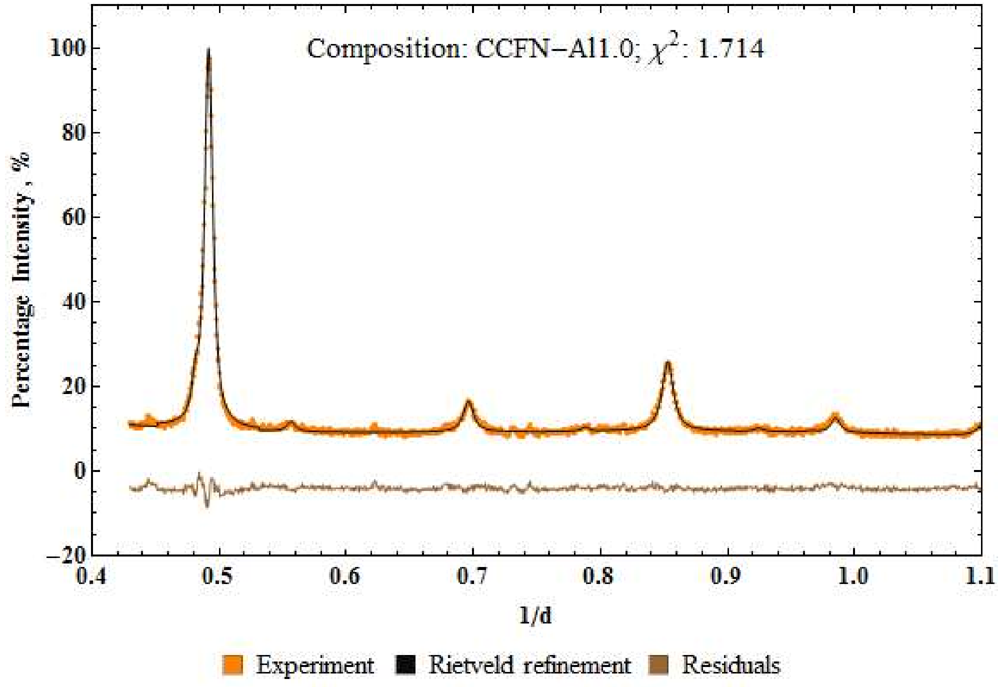

Supplementary Figure S6. CCFN-$Al_{1.0}$ experimental pattern (Orange), Rietveld simulated pattern (Black), and residuals (Brown).

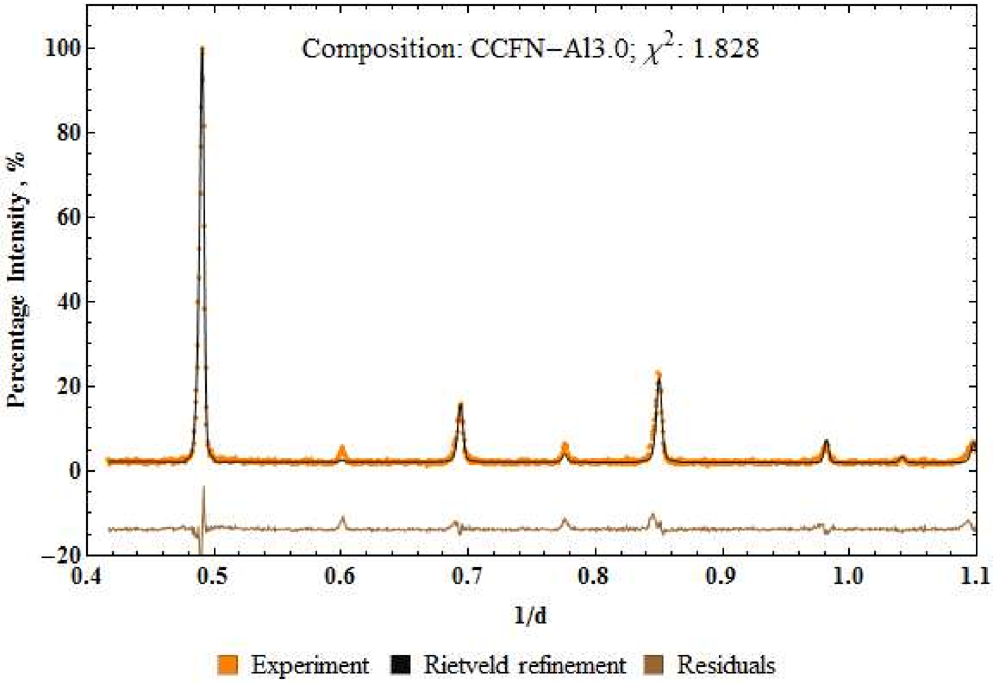

Supplementary Figure S7. CCFN-$Al_{3.0}$ experimental pattern (Orange), Rietveld simulated pattern (Black), and residuals (Brown).

CCFN-V_x systems

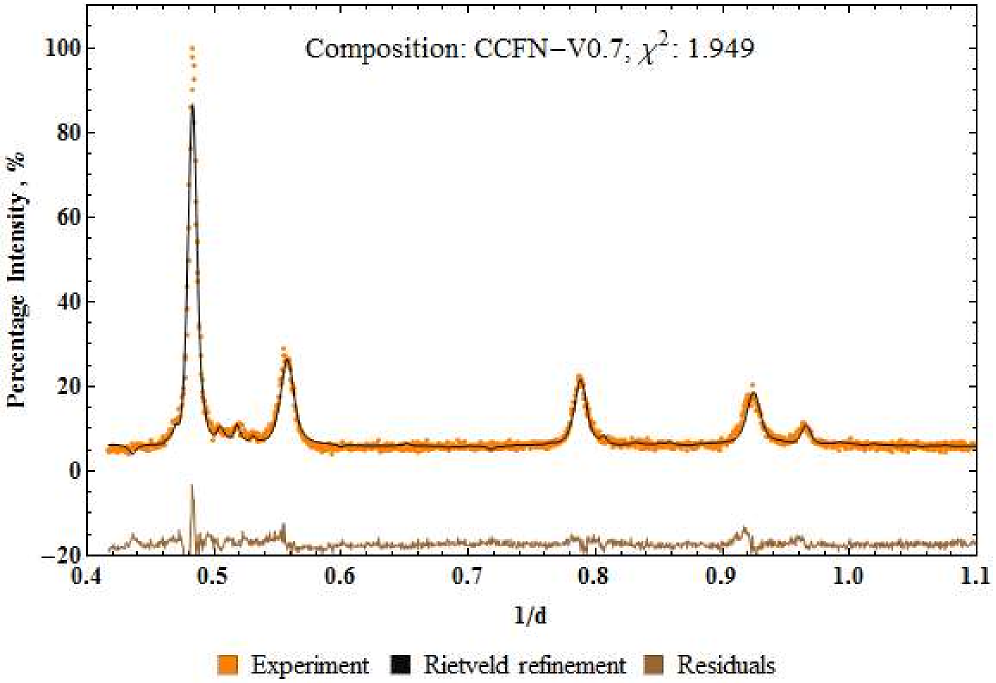

Supplementary Figure S8. CCFN-$V_{0.7}$ experimental pattern (Orange), Rietveld simulated pattern (Black), and residuals (Brown).

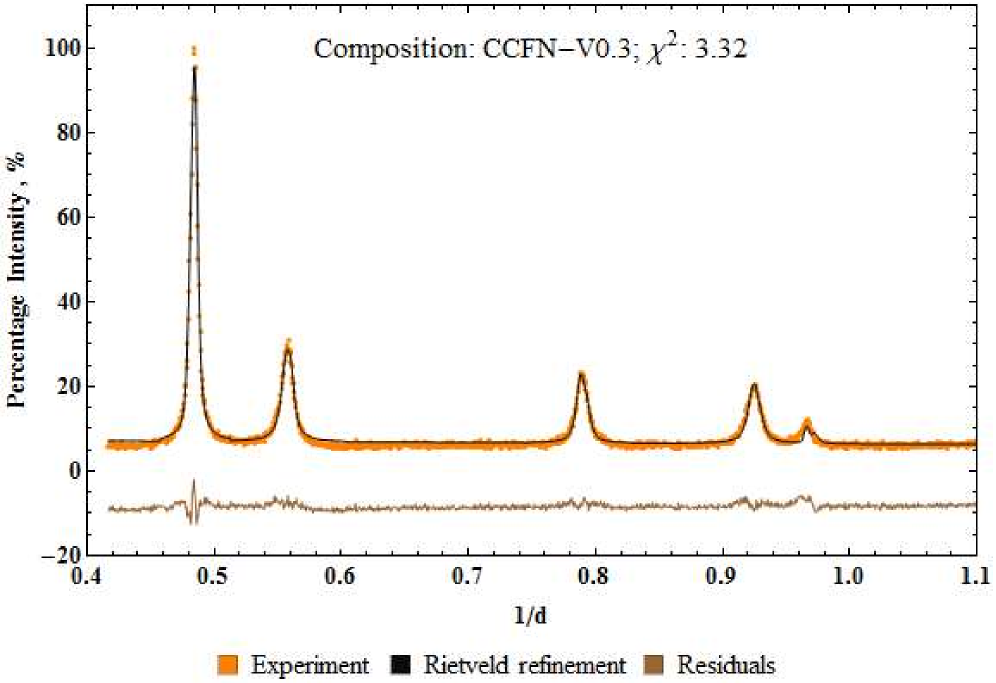

Supplementary Figure S9. CCFN-$V_{0.3}$ experimental pattern (Orange), Rietveld simulated pattern (Black), and residuals (Brown).

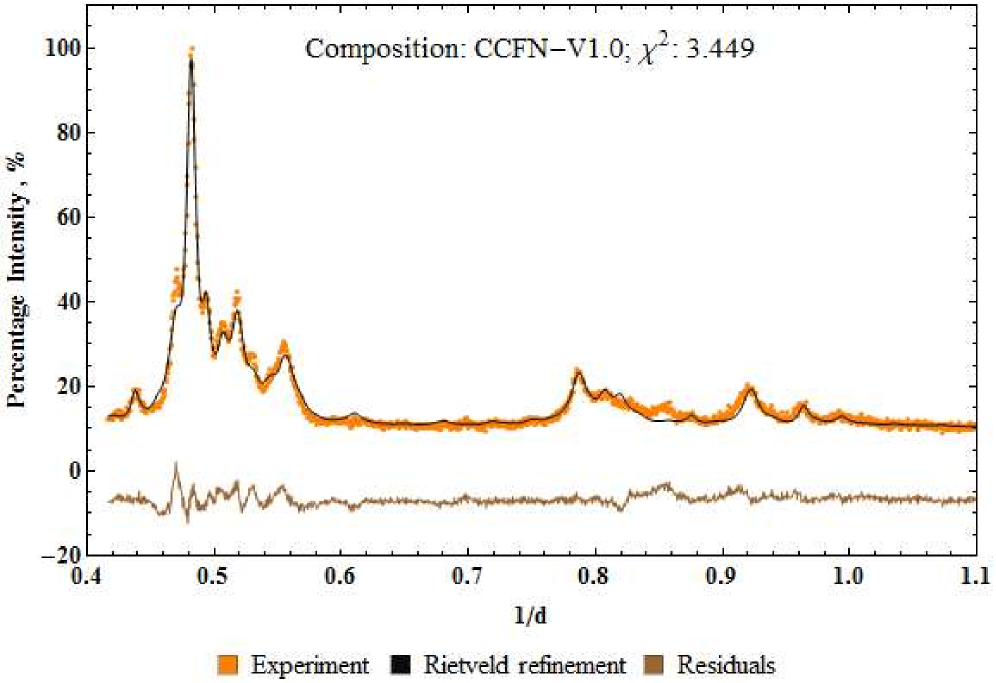

Supplementary Figure S10. CCFN-$V_{1.0}$ experimental pattern (Orange), Rietveld simulated pattern (Black), and residuals (Brown).

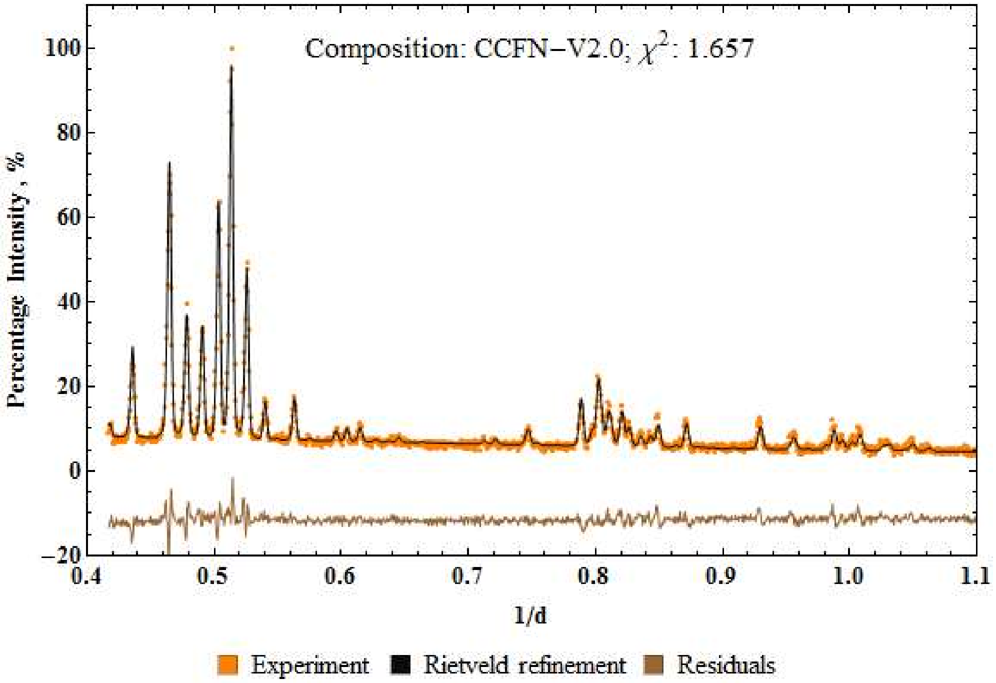

Supplementary Figure S11. CCFN-$V_{2.0}$ experimental pattern (Orange), Rietveld simulated pattern (Black), and residuals (Brown).

CoFN-V_x systems

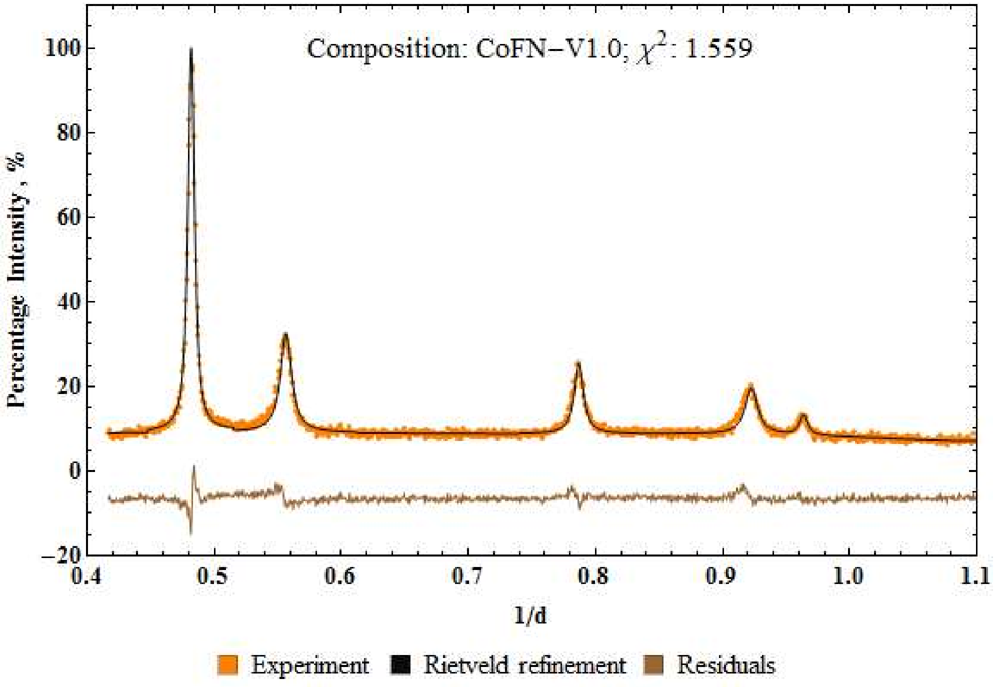

Supplementary Figure S12. CoFN-$V_{1.0}$ experimental pattern (Orange), Rietveld simulated pattern (Black), and residuals (Brown).

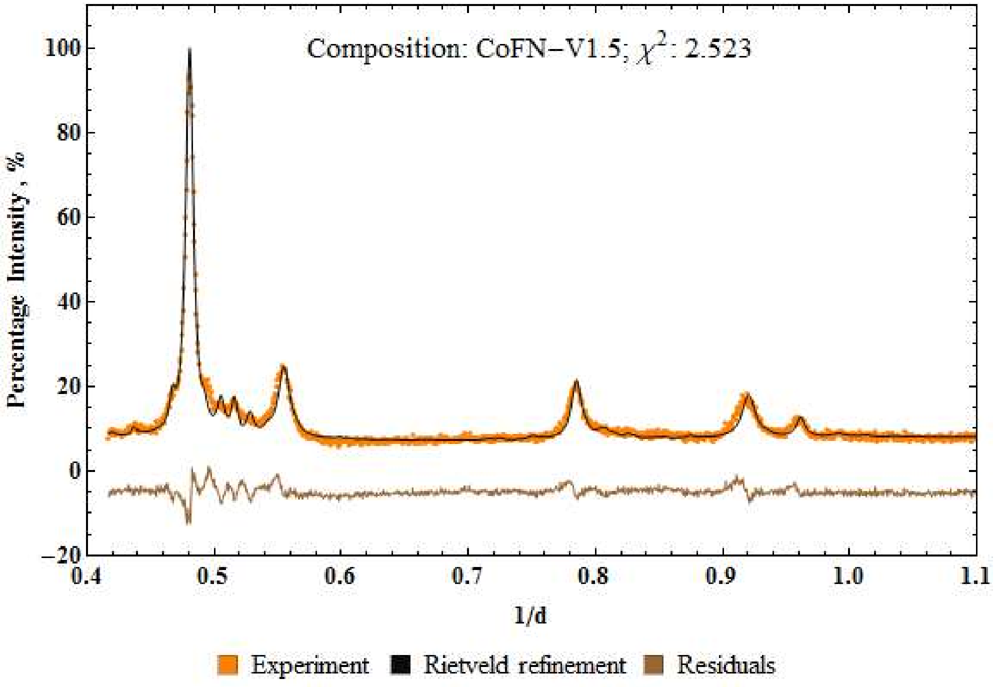

Supplementary Figure S13. CoFN-$V_{1.5}$ experimental pattern (Orange), Rietveld simulated pattern (Black), and residuals (Brown).

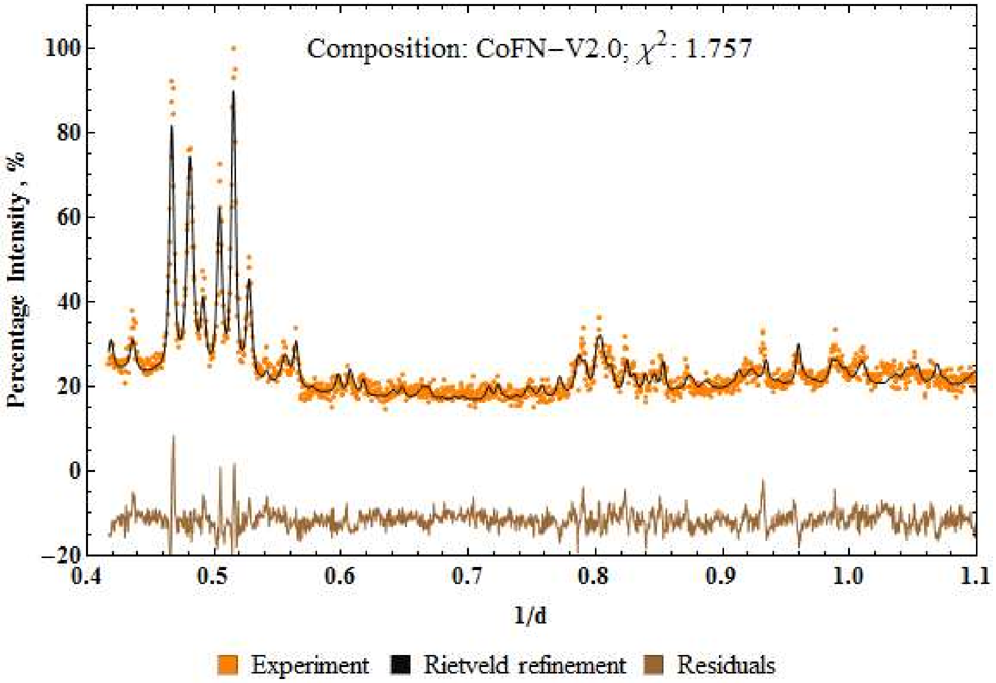

Supplementary Figure S14. CoFN-$V_{2.0}$ experimental pattern (Orange), Rietveld simulated pattern (Black), and residuals (Brown).

CCFN-Ti$_x$ systems

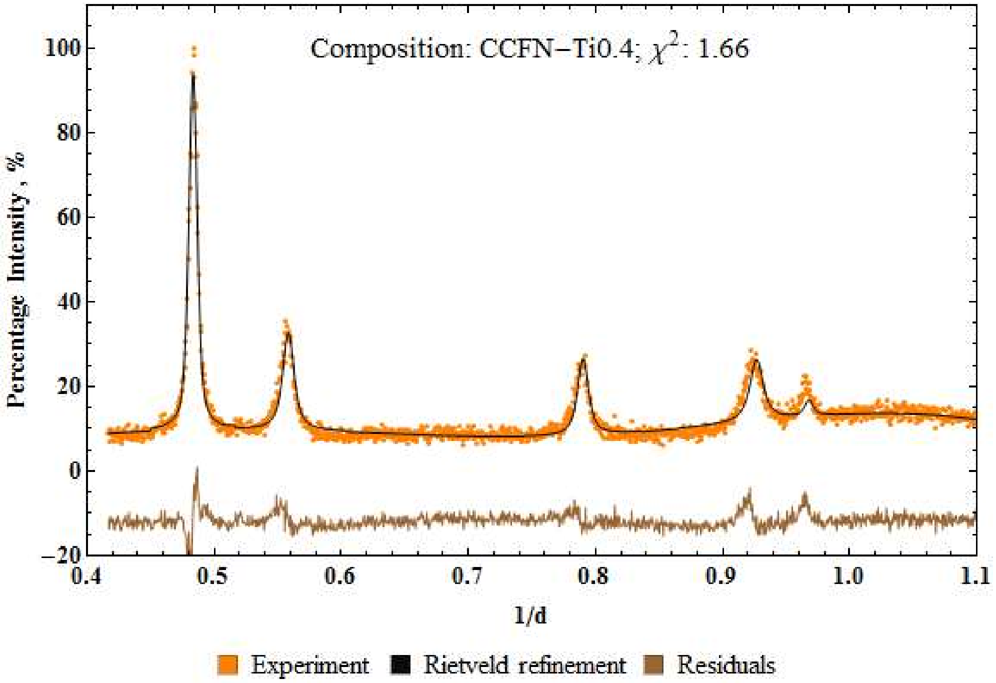

Supplementary Figure S15. CCFN-Ti$_{0.4}$ experimental pattern (Orange), Rietveld simulated pattern (Black), and residuals (Brown).

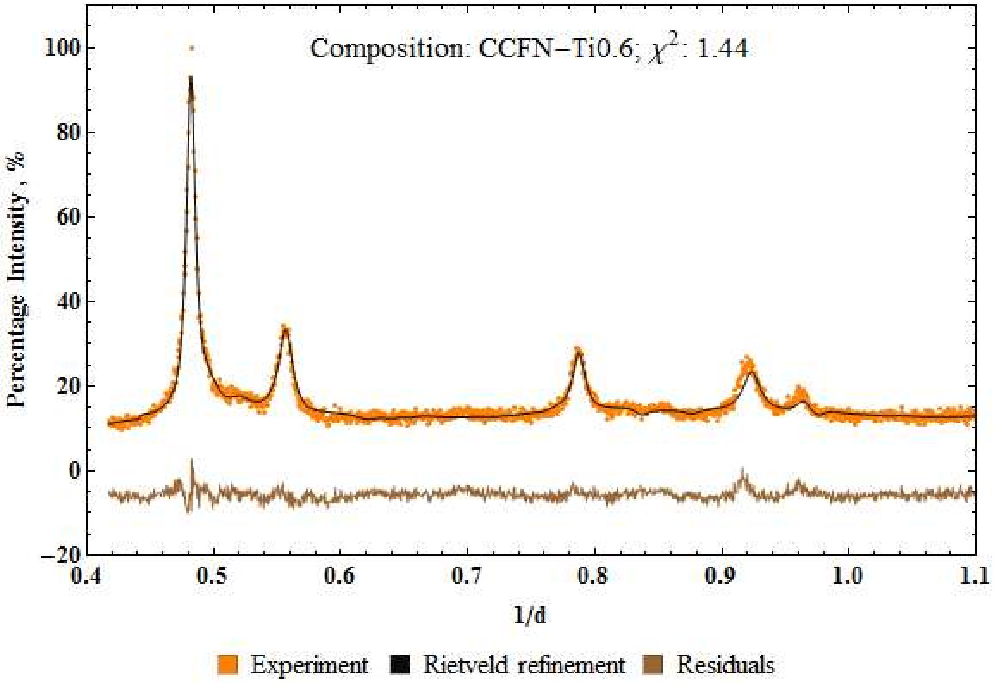

Supplementary Figure S16. CCFN-Ti$_{0.6}$ experimental pattern (Orange), Rietveld simulated pattern (Black), and residuals (Brown).

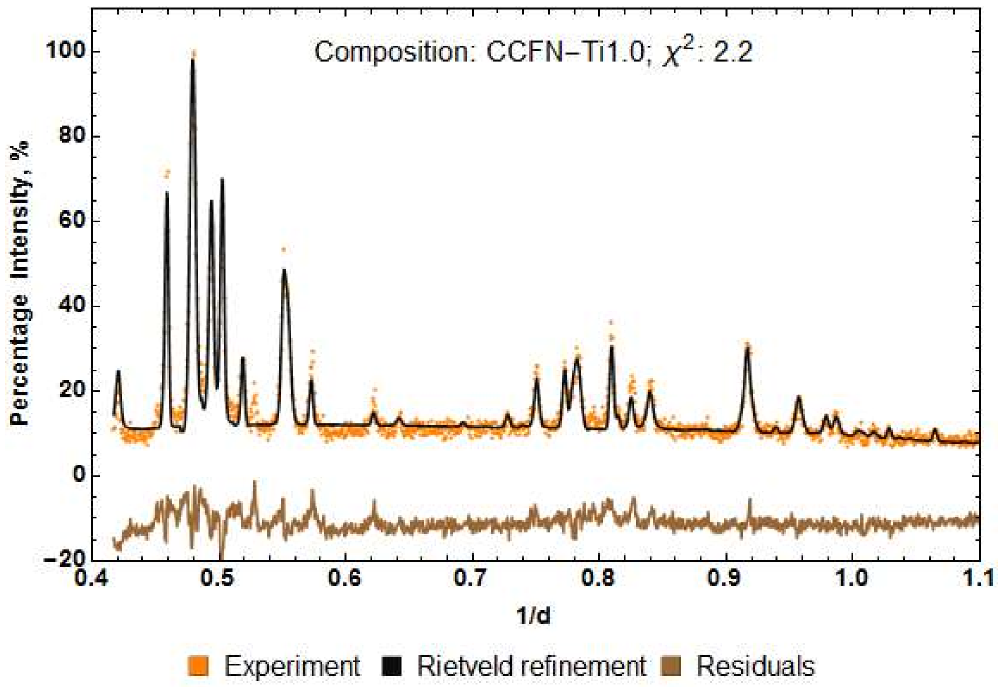

Supplementary Figure S17. CCFN-$Ti_{1.0}$ experimental pattern (Orange), Rietveld simulated pattern (Black), and residuals (Brown).

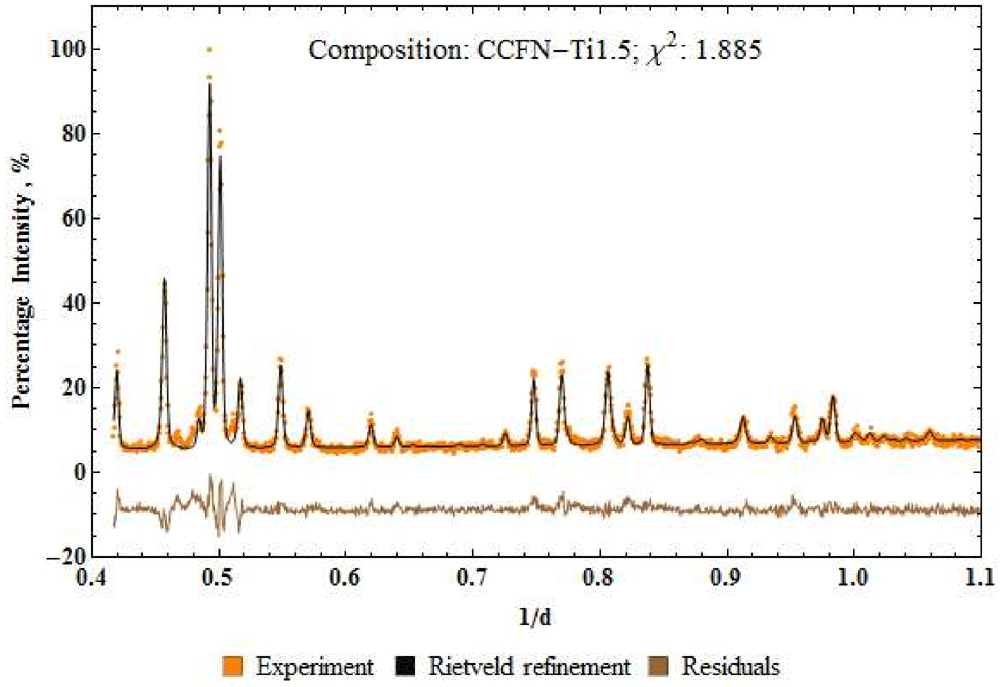

Supplementary Figure S18. CCFN-$Ti_{1.5}$ experimental pattern (Orange), Rietveld simulated pattern (Black), and residuals (Brown).

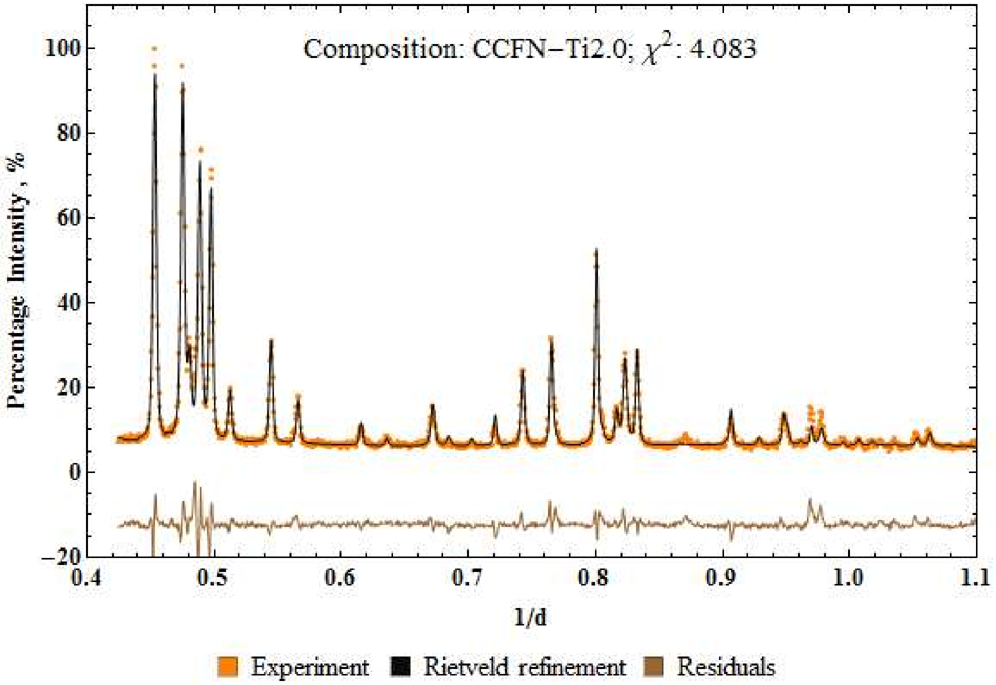

Supplementary Figure S19. CCFN-$Ti_{2.0}$ experimental pattern (Orange), Rietveld simulated pattern (Black), and residuals (Brown).

References

1. Pettifor, D. G. Pressure-cell boundary relation and application to transition-metal equation of state. *Commun. Theor. Phys.* **1,** (1977).
2. Andersen, O. K., Skriver, H. L., Nohl, H. & Johansson, B. J. Electronic structure of transition metal compounds; ground-state properties of the 3d-monoxides in the atomic sphere approximation. *Pure Appl. Chem.* **52,** 93 (1980).
3. Pettifor, D. G. Individual orbital contributions to the SCF virial in homonuclear diatomic molecules. *J. Chem. Phys.* **69,** 2930 (1978).
4. Nguyen-Manh, D., Paxton, A., Pettifor, D. G. & Pasturel, A. On the phase stability of transition metal trialuminide compounds. *Intermetallics* **3,** 9–14 (1995).
5. Paxton, A., Methfessel, M. & Pettifor, D. G. A bandstructure view of the Hume-Rothery electron phases. *Proc. R. Soc. A* **453,** 1493 (1997).
6. Jones, H. J. Concentrated solid solutions of normal metals. **23,** Journal de Physique et le Radium (1962).
7. Liu, G., Nguyen-Manh, D., Liu, B. G. & Pettifor, D. G. Magnetic properties of point defects in iron within the tight-binding-bond Stoner model. *Phys. Rev. B* **71,** 174115 (2005).
8. Nguyen-Manh, D. & Dudarev, S. L. Model many-body Stoner Hamiltonian for binary FeCr alloys. *Phys. Rev. B* **80,** 104440 (2009).
9. Dudarev, S. L. & Derlet, P. M. Interatomic potentials for materials with interacting electrons. *J. Comput.-Aided Mater. Des.* **14,** 129 (2008).
10. Derlet, P. & Dudarev, S. Million-atom molecular dynamics simulations of magnetic iron. *Prog. Mater. Sci.* **52,** 299–318 (2007).
11. Kübler, J. K. *Theory of itinerant electron magnetism*. (Oxford University Press, 2009).
12. Lucas, M. S. *et al.* Magnetic and vibrational properties of high-entropy alloys. *J. Appl. Phys.* **109,** 7 (2011).

13. Christensen, N., Gunnarsson, O., Jepsen, O. & Andersen, O. J. Local spin density theory for ferro- and antiferromagnetic materials. *J. Phys. C* **8,** (1988).
